# HHEX is a transcriptional regulator of the VEGFC/FLT4/PROX1 signaling axis during vascular development

**DOI:** 10.1038/s41467-018-05039-1

**Published:** 2018-07-13

**Authors:** Sébastien Gauvrit, Alethia Villasenor, Boris Strilic, Philip Kitchen, Michelle M. Collins, Rubén Marín-Juez, Stefan Guenther, Hans-Martin Maischein, Nana Fukuda, Maurice A. Canham, Joshua M. Brickman, Clifford W. Bogue, Padma-Sheela Jayaraman, Didier Y. R. Stainier

**Affiliations:** 1 Department of Developmental Genetics, Max Planck Institute for Heart and Lung Research, Bad Nauheim, 61231 Germany; 2 Department of Pharmacology, Max Planck Institute for Heart and Lung Research, Bad Nauheim, 61231 Germany; 3 Institute of Cancer and Genomic Sciences, University of Birmingham, Birmingham, B15 2TT UK; 4 ECCPS Bioinformatics and Deep Sequencing Platform, Max Planck Institute for Heart and Lung Research, Bad Nauheim, 61231 Germany; 5 School of Biological Sciences, Institute for Stem Cell Research, MRC Centre for Regenerative Medicine, Edinburgh, EH16 4UU UK; 6 Novo Nordisk Foundation Centre for Stem Cell Biology (DanStem), University of Copenhagen, Copenhagen, DK-2200 Denmark; 7 Department of Pediatrics, Yale University School of Medicine, New Haven, 06510 CT USA

## Abstract

Formation of the lymphatic system requires the coordinated expression of several key regulators: vascular endothelial growth factor C (VEGFC), its receptor FLT4, and a key transcriptional effector, PROX1. Yet, how expression of these signaling components is regulated remains poorly understood. Here, using a combination of genetic and molecular approaches, we identify the transcription factor hematopoietically expressed homeobox (HHEX) as an upstream regulator of *VEGFC*, *FLT4*, and *PROX1* during angiogenic sprouting and lymphatic formation in vertebrates. By analyzing zebrafish mutants, we found that *hhex* is necessary for sprouting angiogenesis from the posterior cardinal vein, a process required for lymphangiogenesis. Furthermore, studies of mammalian *HHEX* using tissue-specific genetic deletions in mouse and knockdowns in cultured human endothelial cells reveal its highly conserved function during vascular and lymphatic development. Our findings that HHEX is essential for the regulation of the VEGFC/FLT4/PROX1 axis provide insights into the molecular regulation of lymphangiogenesis.

## Abstract (web summary)

VEGFC, its receptor FLT4, and transcriptional effector PROX1 control formation of the lymphatic system but how is unclear. Here, the authors show that the transcription factor hematopoietically expressed homeobox (HHEX) regulates VEGFC, FLT4 and PROX1 in fish and mammals during angiogenic sprouting and lymphatic formation.

## Introduction

Transcriptional regulation of endothelial cell fate and behavior is key to shape and maintain a competent vascular network. During development, lymphatic endothelial cells (LECs) have been reported to arise from a specific subset of vein endothelial cells and require the VEGFC/FLT4/PROX1 signaling axis for their migration, proliferation, and differentiation^1–3^. However, how the expression of these signaling components is regulated remains poorly understood. Of the transcription factor genes regulating endothelial cell physiology, hematopoietically expressed homeobox (*hhex*), also known as proline rich homeodomain (*prh*), is one of the earliest expressed by endothelial and hematopoietic precursors in frogs^4^, zebrafish^5^, and mice^6^. In zebrafish, Hhex can positively regulate endothelial and blood cell differentiation^5^; however, analysis of a deletion allele which lacks *hhex*, as well as several other genes, suggests that Hhex is not essential for early endothelial and blood cell differentiation^5^. In mouse, *Hhex* mutants have multiple developmental defects including marked abnormalities in heart, liver, thyroid, and vascular formation^7,8^. In human endothelial and leukemic cells, HHEX is known to be a direct transcriptional regulator of *VEGFA*, *VEGFR1*, and *VEGFR2*^9^. However, its precise function during angiogenesis and lymphangiogenesis remains unclear.

Through TAL effector nuclease (TALEN) mutagenesis, gene expression analysis, transplantation experiments and endothelial-specific overexpression, we show that in zebrafish, Hhex functions cell-autonomously in endothelial cells to regulate sprouting angiogenesis from the posterior cardinal vein (PCV) and subsequent lymphangiogenesis. We further show through knockdown experiments in vitro and genetic deletions in vivo that this crucial role during angiogenesis and lymphangiogenesis is conserved in mammals. Overall, our data show that HHEX controls blood vessel and lymphatic vessel formation by regulating the VEGFC/FLT4/PROX1 signaling axis.

## Results

### Zebrafish *hhex* mutants exhibit sprouting defects from the PCV

**Fig. 1:**
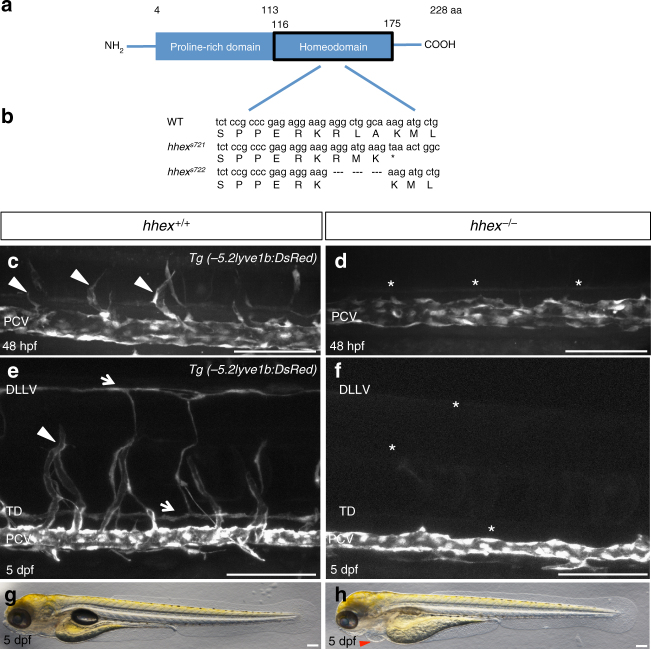
Zebrafish *hhex* mutants lack sprouting angiogenesis from the posterior cardinal vein. **a** Schematic representation of Hhex. Hhex, 228 amino acids (aa) long, is composed of a proline-rich domain (4–113 aa) and a homeodomain (116–175 aa). **b** Alignment of partial Hhex homeodomain sequence in wild-type (WT), and two mutant alleles, *hhex*^*s721*^ and *hhex*^*s722*^. The *hhex*^*s721*^ allele contains a 10 bp insertion leading to a premature stop codon within the homeodomain coding region, whereas the *hhex*^*s722*^ allele lacks amino acids R149 to A151. **c**, **d** Trunk vasculature of *Tg(−5.2lyve1b:DsRed)*; *hhex*^*+/+*^ and *hhex*^*−/−*^ embryos at 48 hpf. *hhex* mutant trunks exhibit a defect in sprouting angiogenesis from the posterior cardinal vein (PCV) (arrowheads point to tip cells sprouting from the PCV; asterisks indicate lack of tip cells sprouting from the PCV). **e**, **f** Trunk vasculature of *Tg(−5.2lyve1b:DsRed)*; *hhex*^*+/+*^ and *hhex*^*−/−*^ larvae at 5 dpf. *hhex* mutant trunks exhibit a defect in the formation of the venous intersegmental vessels (vISVs), the thoracic duct (TD) lymphatic vessel, and the dorsal longitudinal lymphatic vessel (DLLV) (arrowhead points to a vISV; arrows point to the ventrally positioned TD and dorsally positioned DLLV; asterisks indicate lack of these structures). **g**, **h** Brightfield lateral views of *hhex*^*+/+*^ and *hhex*^*−/−*^ larvae at 5 dpf. Mutant larvae exhibit pericardial edema (arrowhead). Scale bars: 100 μm

HHEX is a transcription factor composed of a proline-rich domain and a highly conserved homeodomain^10^. Previously, we used the γ-ray-induced deletion allele *b16* to investigate *hhex* function in zebrafish^10^. However, the *b16* deletion affects the lower telomeric region of chromosome 12 and removes *hhex* and *cyclops* (*ndr2*) as well as at least 14 other genes^11^. *b16* mutants display pleiotropic phenotypes including cyclopia and curvature of the body axis^12^. Therefore, in order to determine more precisely the function of Hhex during zebrafish development, we generated *hhex* mutants using TALENs^13^. TALEN pairs were designed against the homeodomain sequence (Fig. 1a) and two different alleles were recovered: *hhex*^*s721*^ which carries a 10 bp insertion leading to a premature stop codon and *hhex*^*s722*^, an in-frame deletion of three amino acids (R149 to A151) in the second helix of the DNA-binding domain important for the stability of the protein during its interaction with DNA^14^ (Fig. 1b). In all our assays, both alleles exhibit the same phenotype.

In the zebrafish axial vasculature, sprouting angiogenesis occurs in two waves. Sprouting from the dorsal aorta starts at 20 hours post fertilization (hpf) to give rise to arterial intersegmental vessels (aISVs)^15^. Subsequently, endothelial sprouting from the PCV occurs between 32 and 36 hpf^15^ and this process gives rise to both venous ISVs (vISVs) and LECs^16^. aISVs appear to form normally in *hhex* mutants (Supplementary Fig. 1c, d); however, using the *Tg(−5.2lyve1b:DsRed)* line to visualize the venous and lymphatics endothelial cells, we observed that *hhex* mutants lack most sprouting vessels from the PCV (Fig. 1c, d). Time-lapse imaging of wild-type and mutant embryos show that while sprouting angiogenesis from the PCV is clearly observed in wild-type siblings between 32 and 36 hpf, no cell migration from the PCV is visible in *hhex* mutants at least until 48 hpf (Supplementary Movies 1, 2). Furthermore, at 5 days post fertilization (dpf), *hhex* mutants exhibit pericardial edema (Fig. 1g, h), a near absence of vISVs, a complete loss of trunk lymphatic vessels, and a strong reduction of facial lymphatic vessels (Fig. 1e, f, Supplementary Fig. 1e–i, Supplementary Fig. 2). Altogether, these observations indicate that Hhex regulates the earliest step of sprouting angiogenesis and lymphangiogenesis from the PCV.

### Vegfc/Flt4/Prox1 signaling axis is affected in *hhex* mutants

**Fig. 2:**
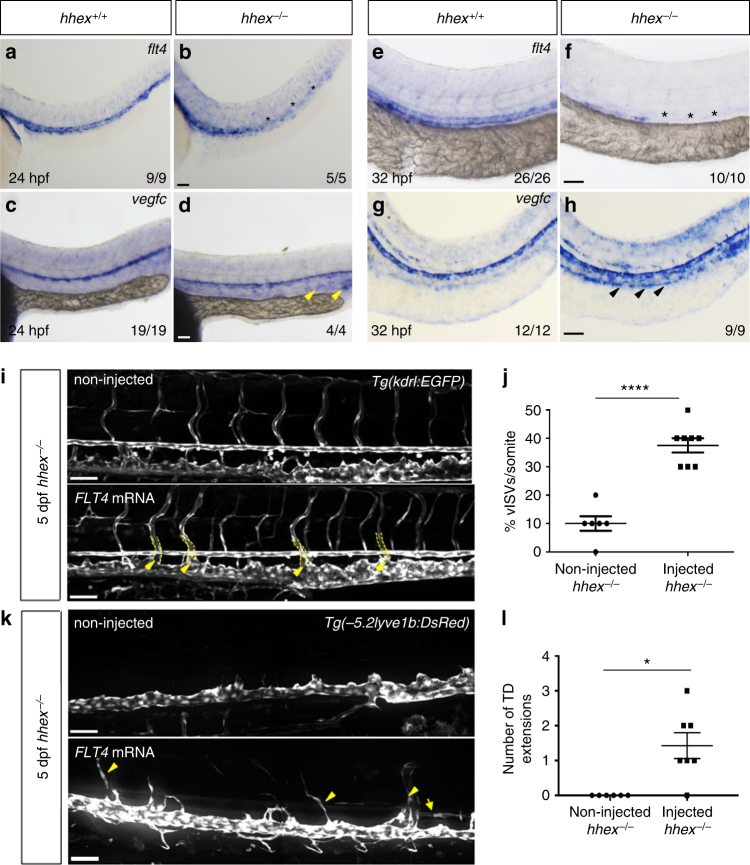
The Vegfc/Flt4 pathway is affected in zebrafish *hhex* mutants. **a**–**d** Whole-mount in situ hybridization showing *flt4* (**a**, **b**) and *vegfc* (**c**, **d**) expression in 24 hpf *hhex*^*+/+*^ and *hhex*^*−/−*^ embryos. At 24 hpf, *hhex* mutants exhibit decreased *flt4* expression (asterisks), whereas *vegfc* expression in the PCV appears to be slightly increased (arrowheads). **e**–**h** Whole-mount in situ hybridization showing *flt4* (**e**, **f**) and *vegfc* (**g**, **h**) expression at 32 hpf in *hhex*^*+/+*^ and *hhex*^*−/−*^ embryos. At 32 hpf, *hhex* mutants exhibit a strong decrease in *flt4* expression (asterisks), whereas *vegfc* expression is clearly increased in the PCV (arrowheads). *x*/*y*: number of embryos showing representative phenotype (*x*), number of embryos examined (*y*). **i** Trunk vasculature of 5 dpf *Tg(kdrl:EGFP); hhex*^*−/−*^ injected, or not, with full-length human *FLT4* mRNA. *hhex* mutants exhibit partial rescue of their vISVs at 5 dpf (arrowheads point to vISVs). **j** Quantification of vISVs across 10 somites in 5 dpf non-injected *hhex*^*−/−*^ (*n* = 6) and *FLT4* mRNA-injected *hhex*^*−/−*^ (*n* = 8). **k** Trunk lymphatic vasculature of 5 dpf *Tg(−5.2lyve1b:DsRed); hhex*^*−/−*^ injected, or not, with full-length human *FLT4* mRNA. *hhex* mutants exhibit partial rescue of their TD at 5 dpf (arrowheads point to vISVs and arrow points to TD). **l** Quantification of TD extensions across 10 somites in 5 dpf non-injected *hhex*^*−/−*^ (*n* = 6) and *FLT4* mRNA-injected *hhex*^*−/−*^ (*n* = 7). Values represent means ± s.e.m. ****P ≤ 0.0001 and *P ≤ 0.05 by *t*-test. Scale bars: 50 μm

Angiogenesis is mostly regulated by vascular endothelial growth factors (VEGFs) and their receptors (VEGFRs). Similar to *hhex* mutants, *vegfc* and *flt4* (also known as *vegfr3*) mutants also fail to initiate sprouting angiogenesis and lymphangiogenesis from the PCV^16–19^. Additionally, *hhex* mutants exhibit a delayed formation of the primordial hindbrain channels at 26 hpf (Supplementary Fig. 1a, b), similar to *vegfc* and *flt4* mutants^17,18^. Therefore, we decided to examine the Vegfc/Flt4 signaling pathway in *hhex* mutants. We first assessed *vegfc* and *flt4* expression at 24 and 32 hpf by whole-mount in situ hybridization (Fig. 2a–h). At 24 hpf, *hhex* mutants exhibit decreased *flt4* expression while *vegfc* was slightly increased and ectopically expressed in the PCV (Fig. 2a–d). These changes were more pronounced at 32 hpf, as *flt4* expression was greatly decreased in *hhex* mutants, whereas *vegfc* expression in the PCV was clearly increased (Fig. 2e–h). To test whether the phenotype we observed in *hhex* mutants might at least in part be due to the modulation of the Vegfc/Flt4 signaling pathway, we performed a rescue experiment by injecting messenger RNA (mRNA) encoding full-length human FLT4 as previously used in zebrafish^18^. Confocal imaging of the trunk vasculature of *hhex* mutant larvae and quantification of vISVs and thoracic duct (TD) extensions revealed a partial rescue of vISVs and lymphatic formation after *FLT4* mRNA injection (Fig. 2i–l), indicating that the Vegfc/Flt4 signaling pathway functions downstream of Hhex. Altogether, these results show that Hhex is a crucial regulator of both *flt4* and *vegfc* expression in the PCV during angiogenic sprouting.

**Fig. 3:**
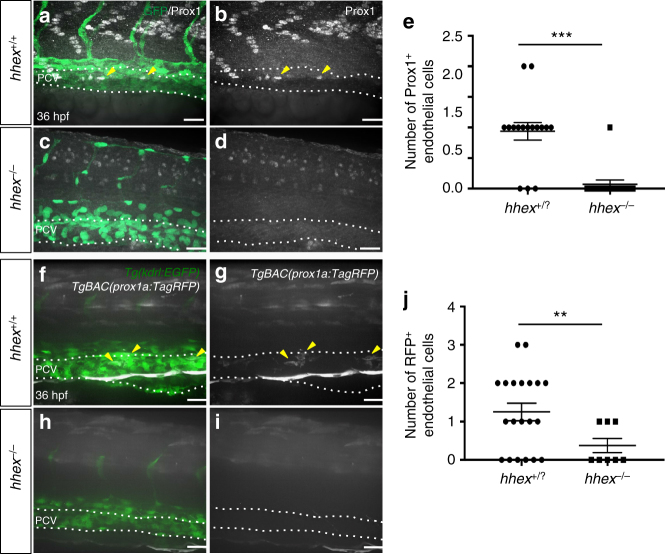
Zebrafish *hhex* mutants lack lymphatic precursors. **a**–**d** Whole-mount views of 36 hpf *hhex*^*+/+*^ and *hhex*^*−/−*^ embryos immunostained for Prox1 (arrowheads point to Prox1^+^ endothelial cells in the PCV). **e** Quantification of the number of Prox1^+^ endothelial cells across three somites (from two field of view per embryo) in *hhex*^*+/?*^ (*n* = 8) and *hhex*^*−/−*^ (*n* = 6). **f**–**i** Whole-mount views of 36 hpf *Tg(prox1a:TagRFP); hhex*^*+/+*^ and *hhex*^*−/−*^ embryos. *hhex* mutants exhibit a strong decrease in the number of RFP^+^ cells in the PCV compared to *hhex*^*+/+*^ (arrowheads point to RFP^+^ endothelial cells in the PCV). **j** Quantification of the number of RFP^+^ endothelial cells across three somites (from two field of view per embryo) in *hhex*^*+/?*^ (*n* = 10) and *hhex*^*−/−*^ (*n* = 4). Values represent means ± s.e.m. ***P ≤ 0.001, and **P ≤ 0.01 by *t*-test. Scale bars: 50 μm

In addition to its role in blood vessel development, the VEGFC/FLT4 signaling axis also plays an instructive role during lymphatic vessel development via activation of the transcription factor PROX1^20,21^. Given that *hhex* mutants display strongly reduced lymphatic formation, we sought to determine whether lymphatic specification was affected in *hhex* mutants by immunostaining for Prox1. In wild-type siblings at 36 hpf, Prox1^+^ endothelial cells are observed in the dorsal part of the PCV. In contrast, *hhex* mutants show a near absence of Prox1^+^ endothelial cells in the dorsal part of the PCV, suggesting that *hhex* mutants lack lymphatic precursors (Fig. 3a–e). To confirm this result, we analyzed the *TgBAC(prox1a:TagRFP)* line which at 36 hpf labels lymphatic precursors prior to their migration from the PCV^20^. Imaging the trunk vasculature of *hhex* mutants at 36 hpf confirmed that the number of lymphatic precursors, as assessed by TagRFP expression, were strongly decreased compared to wild type (Fig. 3f–j).

### Cell-autonomous requirement for Hhex in sprouting angiogenesis

Since *hhex* is expressed not only in endothelial cells but also in endodermal cells^5,22^ and the endoderm has been shown to be important for sprouting angiogenesis from the PCV in zebrafish^23^, we sought to determine in which tissue(s) Hhex was required for angiogenesis. First, we performed whole-mount in situ hybridization for *hhex* expression at 36 hpf, when sprouting angiogenesis starts from the PCV, and at 48 hpf when vISVs have formed. At 36 hpf, *hhex* expression is enriched in budding endothelial cells in the PCV (Supplementary Fig. 3a); in transverse sections, *hhex* expression is observed in the PCV and in the ventral wall of the dorsal aorta (Supplementary Fig. 3b). At 48 hpf, *hhex* is expressed in vISVs and does not appear to be expressed in aISVs based on in situ analysis (Supplementary Fig. 3c). These data indicate that *hhex* is expressed in endothelial cells during sprouting angiogenesis from the PCV.

To determine whether Hhex regulates its own expression, we performed whole-mount in situ hybridization for *hhex* in *hhex*^*s722*^ mutants at 32 hpf. Interestingly, we observed that endothelial *hhex* expression was increased in *hhex*^*−/−*^ compared to wild-type (Supplementary Fig. 4a, b). To test whether the *hhex*^*s722*^ allele encodes a non-functional protein, we injected wild-type and mutant *hhex* mRNA into wild-type zebrafish embryos at the one-cell to four-cell stage (Supplementary Fig. 4c). Overexpression of wild-type *hhex* led to strong developmental defects when injecting 50 to 200 pg while injection of similar amounts of *hhex*^*s722*^ mRNA had little effects on embryonic development (Supplementary Fig. 4c). Altogether, these data indicate that the *hhex*^*s722*^ allele encodes a non-functional protein and that *hhex* can negatively regulate its own expression.

**Fig. 4:**
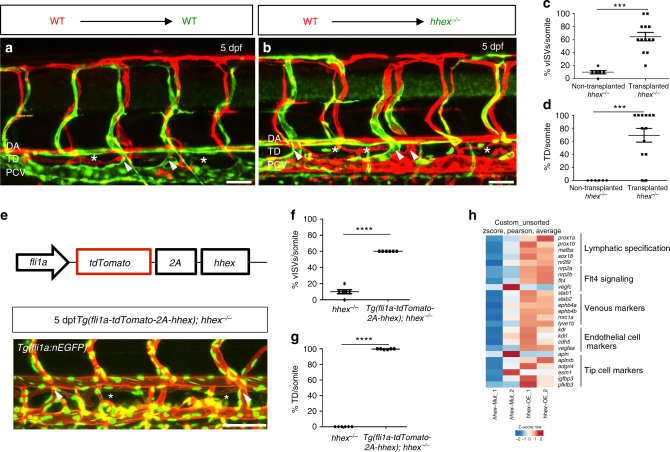
Hhex is required cell-autonomously in endothelial cells to promote venous and lymphatic sprouting in zebrafish. **a**, **b** Transplantation of *Tg(fli1ep:DsRedEx)* donor cells into *TgBAC(etv2:EGFP)* hosts derived from *hhex*^*+/−*^ incrosses. Wild-type endothelial cells contribute to arteries, veins, and lymphatics in wild-type sibling (**a**) and mutant (**b**) hosts at 5 dpf (arrowheads point to vISVs; asterisks indicate TD). **c**, **d** Quantification of vISVs (**c**) and TD extensions (**d**) across four somites in *hhex*^*−/−*^ larvae with transplanted wild-type cells (*n* = 13) vs. *hhex*^*−/−*^ larvae without transplanted wild-type cells (*n* = 6) at 5 dpf. Wild-type endothelial cells can partially rescue both vISV and TD formation in *hhex*^*−/−*^. **e** *hhex* endothelial overexpression strategy using the *fli1a* promoter partially rescues the *hhex*^*−/−*^ vascular phenotype (arrowheads point to vISVs; asterisks indicate TD). **f**, **g** Quantification of vISVs (**f**) and TD (**g**) extensions across four somites in *hhex*^*−/−*^ (*n* = 6) and *Tg(fli1a:tdTomato-2A-hhex); hhex*^*−/−*^ (*n* = 6). **h** RNA sequencing of 48 hpf FACS-sorted *hhex*^*−/−*^ endothelial cells and *hhex*-overexpressing endothelial cells. Heat map comparisons between these datasets identify Hhex as a regulator of genes implicated in lymphatic specification (*prox1a*, *prox1b*, *mafba*, *sox18*, *nr2f2*) and Flt4 signaling (*nrp2a*, *nrp2b*, *flt4*, *vegfc*) while endothelial cell markers are modulated only by endothelial-specific *hhex* overexpression. Values represent means ± s.e.m. ****P ≤ 0.0001 and ***P ≤ 0.001 by *t*-test. Scale bars: 50 μm

To investigate the requirement of *hhex* in endothelial cells to form vISVs and LECs, we generated mosaic animals by transplanting wild-type *Tg(fli1.ep:DsRedEx)* donor cells into *TgBAC(etv2:EGFP)* hosts derived from *hhex*^*+/−*^ incrosses. At 5 dpf, we imaged successfully transplanted larvae and quantified both vISV and TD extensions. We observed that transplanted wild-type endothelial cells were able to contribute to arteries, veins, and lymphatics in wild-type siblings as well as in mutant hosts (Fig. 4a, b and Supplementary Fig. 5). Indeed, transplanted wild-type cells could rescue both vISV and TD formation in *hhex* mutants (Fig. 4c, d). Interestingly, wild-type and *hhex* mutant cells could be observed in vISVs, suggesting a defect in tip cell specification or behavior in *hhex* mutant cells. Wild-type tip cells may thus be sufficient to guide *hhex* mutant stalk cells and assemble a mosaic ISV. However, only wild-type cells were detectable in the TD of *hhex* mutants hosts, indicating that *hhex* is necessary for LEC differentiation.

To further test the endothelial-autonomous requirement of Hhex in sprouting angiogenesis, we overexpressed *hhex* in endothelial cells using the *fli1a* promoter and visualized the transgenic cells by fusing *tdTomato* to the *hhex* coding sequence separated by a self-cleaving viral 2A peptide sequence (Fig. 4e). We generated a stable transgenic line, *Tg(fli1a:tdTomato-2A-hhex)*, in which vascular and lymphatic development appeared to be unaffected (Supplementary Fig. 6) and crossed it to *hhex*^*+/−*^. Incrosses of *Tg(fli1a:tdTomato-2A-hhex); hhex*^*+/−*^ were used to assess and quantify the rescue of the vascular phenotypes at 5 dpf. Quantification of vISV and TD extensions shows that *hhex* expression in endothelial cells was sufficient to rescue both vISV and TD network formation in *hhex* mutants (Fig. 4f, g), thereby confirming the cell-autonomous function of Hhex during the formation of these structures identified in the cell transplantation experiments.

To identify additional Hhex target genes, we performed RNA sequencing on 48 hpf *hhex* mutant endothelial cells, *hhex*-overexpressing endothelial cells, and wild-type endothelial cells (Fig. 4h). Analysis of gene set enrichments between endothelial overexpression and mutant conditions pointed to a role for *hhex* in lymphatic vessel development (upregulation) and hematopoiesis (downregulation) (Supplementary Fig. 7a, b), while comparisons between endothelial overexpression and wild-type conditions pointed to a role for *hhex* in vascular development (upregulation) and hematopoiesis (downregulation) (Supplementary Fig. 7c, d). By comparing endothelial overexpression and mutant conditions, we also found that the expression of genes implicated in lymphatic specification (*prox1a*, *prox1b*, *sox18*, *nr2f2*, *mafba*) and Flt4 signaling (*nrp2a*, *nrp2b*, *flt4*) was modulated by Hhex (Fig. 4h and Supplementary Table 1). Interestingly, endothelial expression of *sox18* and *nr2f2* did not seem to be affected in *hhex* mutants but was upregulated following Hhex overexpression. The expression of venous markers including *stab1*, *stab2*, *ephb4a*, *ephb4b*, and *mrc1a* did not appear to be strongly modulated in *hhex* mutant endothelial cells, except for that of *lyve1b*. As *Hhex* expression has been shown to be enriched in tip cells^24^, we also checked different tip cell markers (*apln*, *aplnrb*, *adgrl4*, *esm1*, *igfbp3*, *pfkfb3*) and found that *hhex* overexpression enhanced *aplnrb* expression but decreased *apln* expression. Finally, using quantitative PCR (qPCR) analysis to further test the RNA sequencing data, we found that *hhex* could regulate *vegfc*, *flt4*, and *prox1a* expression even though *hhex* overexpression was not sufficient to increase *prox1a* expression (Supplementary Fig. 7e). Altogether, these data indicate that Hhex can cell-autonomously regulate the expression of genes implicated not only in lymphangiogenesis but also in sprouting angiogenesis.

### HHEX regulates angiogenesis and lymphangiogenesis in mammals

**Fig. 5:**
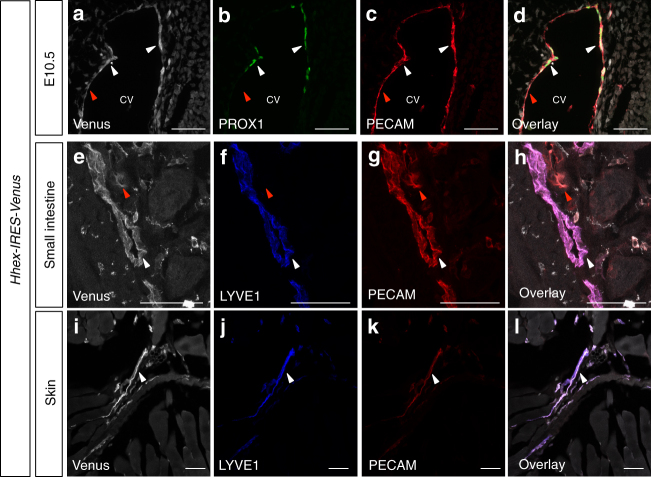
*Hhex-IRES-Venus* is expressed by vascular endothelial cells and lymphatic endothelial cells during mouse development and adulthood. **a**–**d** Maximum intensity projections of confocal images of an E10.5 *Hhex-IRES-Venus* embryo in the CV region after Venus (white), PROX1 (green), and PECAM (red) immunostaining. **e**–**l** Maximum intensity projections of confocal images of an *Hhex-IRES-Venus* adult mouse small intestine (**e**–**h**) and skin (**i**–**l**) after Venus (white), LYVE1 (blue) and PECAM (red) immunostaining. *Hhex-IRES-Venus* expression is observed in blood endothelial cells (red arrowheads) as well as LECs (white arrowheads). Scale bars: 50 μm

To determine whether HHEX function in angiogenesis and lymphangiogenesis is conserved in mammals, we first took advantage of a previously described *Hhex-IRES-Venus* mouse reporter line^25^. We performed immunostaining on transverse sections from embryonic (E) day  E10.5 embryos focusing on the CV region where LEC precursors are still located within the vein and where specified LECs have already migrated outside the vein. We observed *Hhex* reporter expression in PECAM^+^ blood endothelial cells as well as in PECAM^+^/PROX1^+^ LECs (Fig. 5a–d). We also performed immunostaining on adult skin and small intestine and observed that the *Hhex* reporter is still expressed in blood endothelial cells and LECs (Fig. 5e–l). These data show that the *Hhex* reporter is expressed by endothelial cells in both blood and lymphatic vessels, from early development until adulthood.

To understand the precise function of *Hhex* during angiogenesis and lymphangiogenesis, we reanalyzed the phenotype of the global *Hhex* knockout by crossing *Hhex* floxed mice (*Hhex*^*fl/fl*^)^26^ with *Cmv-cre*^27^ to induce recombination in all tissues. We found that *Hhex* mutants were present at a Mendelian ratio at E10.5 and that this ratio decreased starting at E11.5 as previously described^8^. At E10.5, *Hhex* mutant mice exhibited pericardial edema and developmental delay (Supplementary Fig. 8a, b). To characterize the vascular phenotype, we first performed whole-mount PECAM immunostaining on E10.5 embryos and observed strong vascular defects in the head and in the intersomitic vessels in the trunk (Supplementary Fig. 8c–f). We also analyzed the CV region and found a decreased number of PROX1^+^ LECs within the CV and these cells appeared to exhibit weaker PROX1 immunostaining than in wild-types (Supplementary Fig. 8g–i). At E14.5, only 12.5% of *Hhex* mutants could be recovered (mostly as dead or resorbed embryos), and the few surviving *Hhex* mutants presented strong vascular defects and blood-filled lymphatics (Supplementary Fig. 7j, k).

**Fig. 6:**
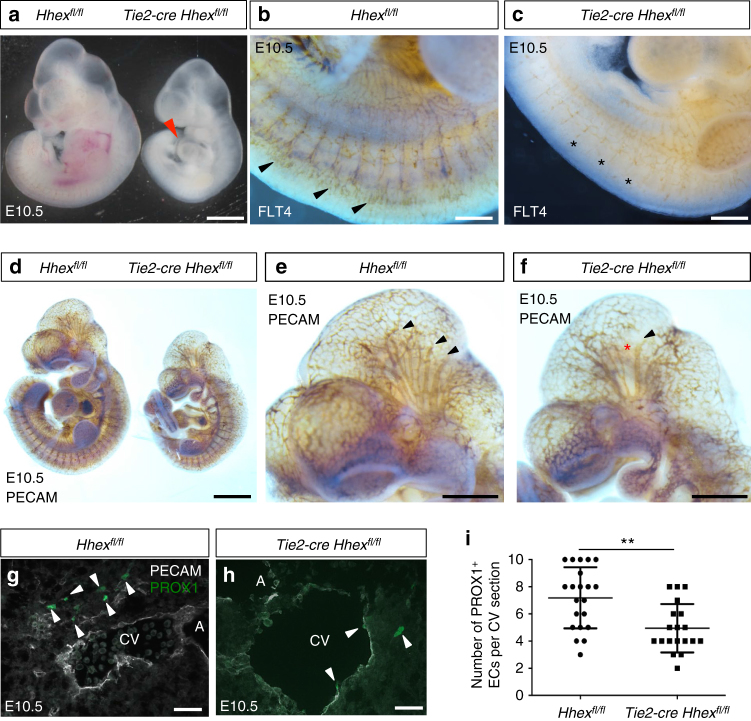
*Tie2-cre Hhex*^*fl/fl*^ mouse embryos exhibit minor vascular defects and a reduced number of PROX1^+^ endothelial cells at E10.5. **a** Whole-mount view of *Hhex*^*fl/fl*^ and *Tie2-cre Hhex*^*fl/fl*^ embryos at E10.5. *Hhex* mutants exhibit pericardial edema (red arrowhead) as well as developmental delay. **b**, **c** Whole-mount views of *Hhex*^*fl/fl*^ and *Tie2-cre Hhex*^*fl/fl*^ embryos at E10.5 after FLT4 immunostaining. *Hhex* mutants exhibit a strong decrease in FLT4 expression in the intersomitic vessels (arrowheads point to intersomitic vessels; asterisks indicate reduction of FLT4 expression in the same structures). **d**–**f** Whole-mount view of *Hhex*^*fl/fl*^ and *Tie2-cre Hhex*^*fl/fl*^ embryos at E10.5 after PECAM immunostaining. *Hhex* mutants exhibit minor vascular defects in the head (arrowheads point to blood vessels in *Hhex*^*fl/fl*^ and *Tie2-cre Hhex*^*fl/fl*^; asterisk indicates lack of vessels in the same structures in *Tie2-cre Hhex*^*fl/fl*^). **g**, **h** Maximum intensity projections of confocal images from transverse cryosections of *Hhex*^*fl/fl*^ and *Tie2-cre Hhex*^*fl/fl*^ E10.5 embryos after immunostaining for PECAM (white) and PROX1 (green) in the cardinal vein (CV) and aorta (A) region. *Hhex* mutants exhibit fewer PROX1^+^/PECAM^+^ endothelial cells in the region of the CV (arrowheads point to PROX1^+^/PECAM^+^ endothelial cells). **i** Quantification of the number of PROX1^+^ endothelial cells in the CV region in *Hhex*^*fl/fl*^ (*n* = 2) and *Tie2-cre Hhex*^*fl/fl*^ (*n* = 2) embryos. Values represent means ± s.e.m. **P ≤ 0.01 by *t*-test. Scale bars: 1 mm (**a**, **d**), 200 μm (**b**, **c**), 500 μm (**e**, **f**), and 20 μm (**g**, **h**)

As the strong vascular defects we observed in *Hhex* mutants can affect the number of lymphatic precursors, we decided to cross *Hhex*^*fl/fl*^ with *Tie2-cre* mice^28^ which carry a *cre* transgene active in the blood islands at early stages and targeting both endothelial and hematopoietic cells^29^. No *Tie2-cre Hhex*^*fl/fl*^ animals were recovered at birth, suggesting that mice lacking *Hhex* in *Tie2*-expressing cells die during embryogenesis. At E10.5, *Tie2-cre Hhex*^*fl/fl*^ embryos were recovered at a Mendelian ratio and, similarly to what was observed with the global *Hhex* mutants, this ratio decreased starting at E11.5. *Tie2-cre Hhex*^*fl/fl*^ embryos exhibited pericardial edema and developmental delay (Fig. 6a). Similarly, *Flt4* mutant embryos started to die from E10.5 due to severe cardiovascular defects and exhibited yolk sac vasculature defects, pericardial edema, and developmental delay^30^. We performed whole-mount FLT4 immunostaining on E10.5 embryos and observed that *Tie2-cre Hhex*^*fl/fl*^ embryos exhibited a strong decrease in FLT4 immunostaining notably in the intersomitic vessels (Fig. 6b, c), possibly explaining the vascular phenotype. To further characterize the vascular phenotype, we also performed whole-mount PECAM immunostaining on E10.5 embryos and observed some vascular branching defects in the head (Fig. 6d–f).

**Fig. 7:**
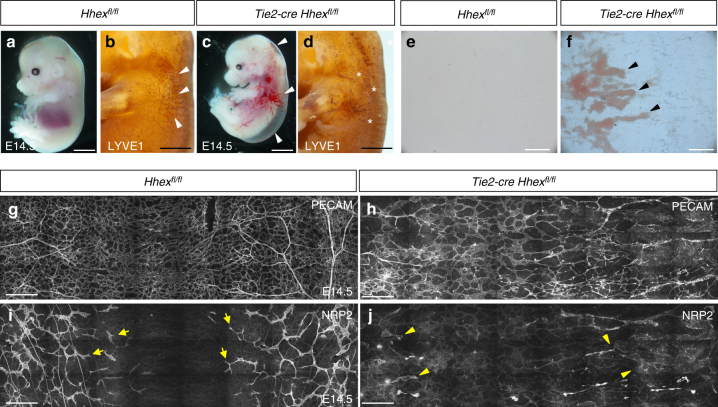
*Tie2-cre Hhex*^*fl/fl*^ mouse embryos exhibit strong vascular and lymphatic defects at E14.5. **a**–**c** Morphology of *Hhex*^*fl/fl*^ and *Tie2-cre Hhex*^*fl/fl*^ mouse embryos at E14.5. *Hhex* inactivation in *Tie2*-expressing cells leads to lymphatic defects including edema and blood-filled lymphatics (arrowheads). **b**–**d** Whole-mount views of *Hhex*^*fl/fl*^ and *Tie2-cre Hhex*^*fl/fl*^ embryos at E14.5 after LYVE1 immunostaining. *Hhex* mutants exhibit defects in lymphatic patterning with a reduced lymphatic vasculature in the skin (arrowheads indicate LYVE1^+^ lymphatic vessels, asterisks indicate lack of these vessels in the same area in *Tie2-cre Hhex*^*fl/fl*^). **e**, **f** Brightfield pictures of *Hhex*^*fl/fl*^ and *Tie2-cre Hhex*^*fl/fl*^ skin at E14.5. *Hhex* mutants exhibit blood-filled lymphatics (arrowheads). **g**, **h** Whole-mount views of *Hhex*^*fl/fl*^ and *Tie2-cre Hhex*^*fl/fl*^ skin at E14.5 after PECAM immunostaining. *Hhex* mutants exhibit strong vascular defects at E14.5. **i**, **j** Whole-mount views of *Hhex*^*fl/fl*^ and *Tie2-cre Hhex*^*fl/fl*^ skin at E14.5 after NRP2 immunostaining. *Hhex* mutants exhibit strong lymphatic defects at E14.5 (arrows point to lymphatic vessels; arrowheads point to blood-filled lymphatic vessels with decreased intensity of NRP2 immunostaining). Scale bars: 2 mm (**a**, **c**), 1 mm (**b**, **d**), 200 μm (**e**, **f**), and 250 μm (**g**–**j**)

The VEGFC/FLT4/PROX1 signaling axis has been shown to regulate the number of LECs in a dose-dependent manner^21,31,32^. To investigate whether HHEX affects the number of LECs, we quantified the number of PROX1^+^ cells in the CV region. *Tie2-cre Hhex*^*fl/fl*^ mutants contained fewer PROX1^+^ endothelial cells in the CV compared to wild-type siblings (Fig. 6g–i). Importantly, and as observed in global *Hhex*^*−/−*^ embryos, these LECs were mostly within the vein and rarely outside. As decreased expression levels of *Flt4* and *Prox1* have been shown to trigger the same phenotype, these results suggest a migration defect in addition to the observed reduction in the number of LECs. At E14.5, the percentage of *Tie2-cre Hhex*^*fl/fl*^ embryos recovered was below the expected Mendelian ratio (around 15% instead of the expected 25%), indicating that some *Tie2-cre Hhex*^*fl/fl*^ embryos die at earlier stages. *Tie2-cre Hhex*^*fl/fl*^ embryos alive at this stage exhibited severe lymphatic defects with edema and blood-filled lymphatics (Fig. 7a, c, e-f). Moreover, whole-mount LYVE1 immunostaining revealed that *Tie2-cre Hhex*^*fl/fl*^ embryos exhibit defects in the superficial lymphatic vasculature including a reduction in the number of lymphatic vessels (Fig. 7b, d). We also examined the effects of deleting *Hhex* in *Tie2-Cre*-expressing cells on the blood and lymphatic vasculature using the dorsal skin model. At E14.5, LECs sprout and migrate towards the dorsal midline to form a stereotypical network^33^. Whole-mount immunostaining of the embryonic mouse skin with PECAM and NRP2 antibodies showed that both blood and lymphatic vascular networks were strongly affected in *Tie2-cre Hhex*^*fl/fl*^ embryos compared to *Hhex*^*fl/fl*^ (Fig. 7g–j). Interestingly, lymphatic vessels in *Tie2-cre Hhex*^*fl/fl*^ mutants displayed decreased intensity of NRP2 immunostaining compared to wild-type.

**Fig. 8:**
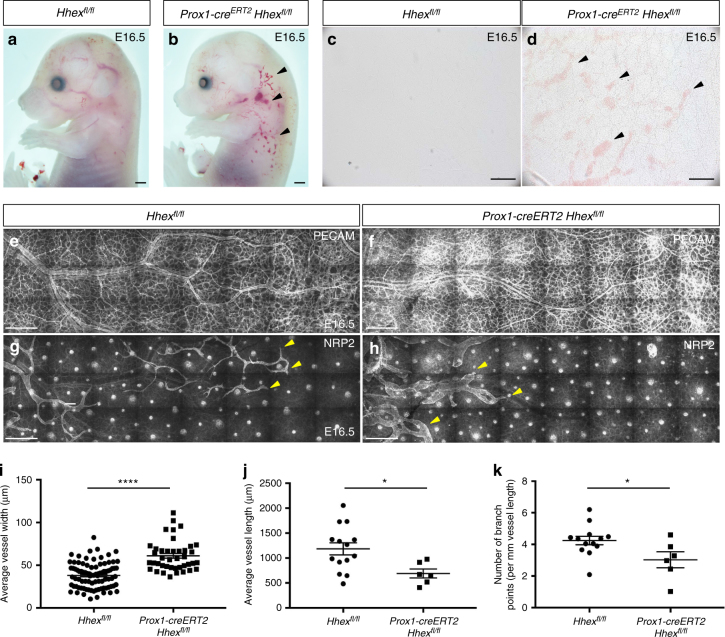
*Prox1-cre*^*ERT2*^ *Hhex*^*fl/fl*^ mouse embryos exhibit lymphatic defects at E16.5. **a**, **b** Whole-mount views of *Hhex*^*fl/fl*^ and *Prox1-cre*^*ERT2*^ *Hhex*^*fl/fl*^ embryos at E16.5 after tamoxifen injection at E10.5, 11.5, and 12.5. *Hhex* deletion in *Prox1*-expressing cells leads to lymphatic defects including edema and blood-filled lymphatics (arrowheads). **c**, **d** Brightfield pictures of *Hhex*^*fl/fl*^ and *Prox1-cre*^*ERT2*^ *Hhex*^*fl/fl*^ skin at E16.5. *Hhex* mutants exhibit blood-filled lymphatics (arrowheads). **e**–**h** Whole-mount views of *Hhex*^*fl/fl*^ and *Prox1-cre*^*ERT2*^ *Hhex*^*fl/fl*^ skin at E16.5 after PECAM (**e**–**f**) and NRP2 (**g**, **h**) immunostaining. *Hhex* mutants do not exhibit vascular defects but clear lymphatic defects (arrowheads point to lymphatic vessels). **i**–**k** Quantification of the lymphatic network including average vessel width (**i**), average vessel length (**j**), and number of branch points (per mm vessel length) (**k**) in *Hhex*^*fl/fl*^ (*n* = 3) and *Prox1-cre*^*ERT2*^ *Hhex*^*fl/fl*^ (*n* = 3) skin at E16.5. Values represent means ± s.e.m. ****P ≤ 0.0001 and *P ≤ 0.05 by *t*-test. Scale bars: 1 mm (**a**, **b**), 200 μm (**c**, **d**), and 250 μm (**e**–**h**)

**Fig. 9:**
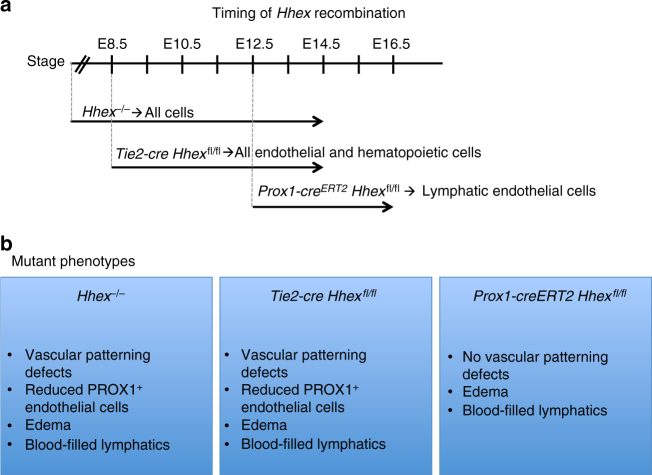
Summary of the effects of global and conditional *Hhex* deletion during vascular development in mouse. **a** Stages when *Hhex* recombination occurs in *Hhex*^*−/*^^*−*^, *Tie2-cre Hhex*^*fl/fl*^, and *Prox1-cre*^*ERT2*^ *Hhex*^*fl/fl*^. **b** Consequences of *Hhex* deletion in *Hhex*^*-/-*^, *Tie2-cre Hhex*^*fl/fl*^, and *Prox1-cre*^*ERT2*^ *Hhex*^*fl/f*^ are summarized in the blue boxes

In order to investigate the role of *Hhex* during lymphatic development, we crossed *Hhex*^*fl/fl*^ with *Prox1-cre*^*ERT2*^, a tamoxifen-inducible cre recombinase expressed in LECs^34^. Tamoxifen delivery from E10.5 to 12.5 to delete *Hhex* in LECs after their specification resulted in edema and blood-filled lymphatics at E16.5 (Fig. 8a–d). Additionally, 10% of *Prox1-cre*^*ERT2*^ *Hhex*^*fl/fl*^ embryos were found dead at this stage. Whole-mount immunostaining of the embryonic mouse skin with PECAM and NRP2 antibodies showed that the blood vascular network was not affected in *Prox1-cre*^*ERT2*^ *Hhex*^*fl/fl*^ embryos, while the lymphatic network was clearly affected (Fig. 8e–h). The lymphatic vessels were shorter, wider, and exhibited fewer branches in mutants compared to wild-types (Fig. 8i–k). These results show that *Hhex* is important for the establishment of the lymphatic network during dermal lymphatic formation. Altogether, we investigated the role of *Hhex* during blood and lymphatic vascular development using global and conditional mouse mutants. The timing of *Hhex* recombination as well as the different phenotypes observed in *Hhex*^*−/−*^, *Tie2-cre Hhex*^*fl/fl*^, and *Prox1-cre*^*ERT2*^ *Hhex*^*fl/fl*^ embryos are summarized in Fig. 9.

**Fig. 10:**
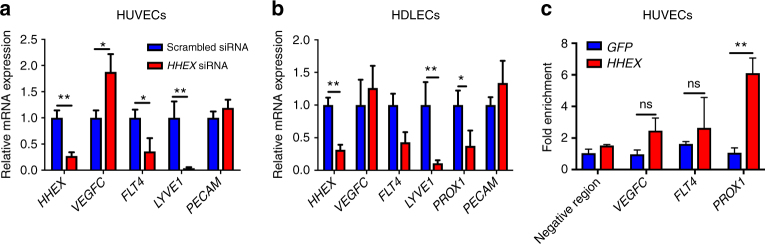
HHEX regulates the VEGFC/FLT4/PROX1 pathway in human endothelial cells and binds the PROX1 promoter. **a**, **b** qPCR analysis of *HHEX*, *VEGFC*, *FLT4*, *LYVE1*, *PROX1*, and *PECAM* expression in HUVECs (**a**) and HDLECs (**b**) treated with *HHEX* siRNA compared to control cells treated with a scrambled siRNA. Knockdown of *HHEX* led to downregulation of *FLT4*, *PROX1*, and *LYVE1* and upregulation of *VEGFC* expression. **c** Chromatin immunoprecipitation (ChIP) experiment using *Myc-HHEX* adenovirus in HUVECs. qPCR using primers for specific *FLT4*, *VEGFC*, and *PROX1* regions was carried out and enrichment relative to the percentage of input in the IP sample at *PRM2* (control region) is shown. HHEX does not appear to bind the *FLT4* promoter or *VEGFC* 3′ region but strongly binds the *PROX1* promoter. Values represent means ± s.e.m. **P ≤ 0.01; *P ≤ 0.05; ns, not significant by Mann-Whitney (**a**, **b**) or Bonferroni post hoc (**c**)

To test whether the role of HHEX is conserved in humans, we performed cell culture experiments to knockdown *HHEX* in human umbilical vein endothelial cells (HUVECs) (Fig. 10a) and in human dermal LECs (HDLECs) (Fig. 10b). Similarly to what we observed in both zebrafish and mouse embryos, decrease of *HHEX* expression in HUVECs and HDLECs led to decreased expression of *FLT4* while *VEGFC* was upregulated (Fig. 10a, b). Interestingly, *HHEX* knockdown in HDLECs also led to downregulation of *PROX1* expression consistent with our observations in zebrafish and mouse (Fig. 10b).

Using in silico predictions, we identified putative HHEX binding sites 2 kb upstream of the *FLT4* transcriptional start site, 800 bp upstream of the *PROX1* transcriptional start site, and in the 3′ region of *VEGFC*. To test whether the transcriptional changes we observed were a result of direct HHEX binding to *VEGFC*, *FLT4*, and *PROX1* regulatory elements or via indirect routes, we performed chromatin immunoprecipitation (ChIP) experiments using *Myc-HHEX* adenovirus transfected in HUVECs (Fig. 10c). We observed a significant enrichment of Myc-HHEX at the putative HHEX binding sites upstream of the *PROX1* transcriptional start site (Fig. 10c), suggesting that *PROX1* is a direct transcriptional target of HHEX. In contrast, our analyses did not show binding of HHEX directly to potential regulatory regions of *FLT4* or *VEGFC* (Fig. 10c), suggesting that HHEX regulation of these targets is indirect, or that other HHEX binding sites are present in distal regions. Altogether, these data indicate that HHEX regulates the VEGFC/FLT4/PROX1 axis in zebrafish, mouse, and human endothelial cells.

## Discussion

Although the transcriptional networks underlying the development of the blood and lymphatic vessels have started to become better described, a complete understanding of these processes remains a major goal. Here, using a combination of systems and approaches, we have shown that the transcription factor HHEX functions upstream of several key players in blood and lymphatic vessel development, namely those of the VEGFC/FLT4/PROX1 axis.

The formation of the lymphatic vasculature through the VEGFC/FLT4/PROX1 axis is an evolutionarily conserved process across vertebrates. Here, using zebrafish, we showed that *hhex* is necessary for venous and lymphatic sprouting from the PCV. *hhex* mRNA expression is not restricted to lymphatic precursors as marked by Prox1 expression, but is observed in both venous endothelial cells and LECs during sprouting from the PCV. *hhex* mutants exhibit unique gene expression changes, with a strong decrease in *flt4* expression coupled to ectopic expression of *vegfc* in the PCV. Interestingly, *FLT4* overexpression in zebrafish *hhex* mutants did not completely rescue the angiogenic defects. It is possible that the loss of *hhex* function affects additional pathways involved in sprouting angiogenesis from the vein, or that the *FLT4* mRNA injections are not able to provide adequate levels of Flt4 function when needed. Furthermore, overexpression of *hhex* in endothelial cells increased *flt4* expression while *vegfc* was downregulated. One possible explanation for these observations is that reduction of *flt4* expression could affect *vegfc* expression and vice versa. However, Le Guen et al.^19^ showed that *flt4* level was not affected in *vegfc* morphants (and vice versa), thus indicating that Vegfc and Flt4 do not cross-regulate each other transcriptionally. Thus, Hhex appears to regulate at the same time and in an opposite fashion the transcription of genes encoding a growth factor and its receptor.

HHEX has also been implicated in a number of human pathologies, with a crucial role in several types of cancer^35,36^. *HHEX* is overexpressed in human acute myeloid leukemia^35^, while in breast cancer HHEX functions as a suppressor of tumor growth and negatively regulates *VEGFC*^37^. Additionally, *HHEX* expression is upregulated in dermal LECs from type 2 diabetes patients, suggesting a potential lymphatic role for *HHEX* in pathological conditions^38^.

Our RNA sequencing data suggest that Hhex can negatively regulate its own expression in endothelial cells. Using in situ hybridization, we indeed observed that endothelial *hhex* expression was increased in *hhex* mutants. This finding is consistent with a recent report in hematopoietic cells^39^.

PROX1 is one of the key transcription factors driving the fate and specification of LECs in all vertebrates. We found that *hhex* mutants lack lymphatic precursors as assessed by Prox1 immunostaining and *TgBAC(prox1a:*TagRFP*)* expression. Interestingly, PROX1 is expressed in many of the same tissues and developmental stages as HHEX, including hepatoblasts^22^. Here, we show that HHEX and PROX1 are co-expressed in developing LECs and that HHEX is necessary for PROX1 mRNA and protein expression. RNA sequencing data of endothelial cells from whole zebrafish embryos indicate that *hhex* overexpression does not increase *prox1a* expression, suggesting that Hhex co-factors present in subsets of endothelial cells might be necessary to drive *prox1* expression. *Hhex* and *Prox1* are also both expressed in endodermal tissues in zebrafish^40^ and mouse^41^ embryos, and it will be interesting to analyze in detail the expression pattern of these genes in wild-type and mutant animals to better understand how these genes regulate and maintain the differentiation of various cell types including LECs.

Our data show that Hhex is required cell-autonomously in endothelial cells to promote venous and lymphatic sprouting. Using transplants to generate genetic mosaics, we found that tip cells in mutant hosts were always wild-type while *hhex* mutant cells could participate in vISV formation, suggesting that Flt4 signaling is not uniformly required for vISV formation but only for tip cell identity and behavior. This observation has also been reported in other sprouting angiogenesis contexts including Wnt signaling and brain angiogenesis^42^. Additionally, we observed that only wild-type cells could be found in the TD indicating a strict cell-autonomous requirement for Hhex in LEC formation in zebrafish.

Using different mouse models, we show that *Hhex* is important for angiogenesis and lymphangiogenesis. *Hhex*^*−/−*^ embryos exhibit severe developmental defects in blood and lymphatic vessel development while *Hhex* deletion in a more specific cell population using the *Tie2-cre* driver recapitulates the global knockout phenotype. We found that *Tie2-cre Hhex*^*fl/fl*^ embryos exhibit a strong decrease in FLT4 immunostaining at E10.5, possibly explaining the vascular phenotypes. We also observed a reduced number of Prox1^+^ cells within the cardinal vein at E10.5, and these cells had not moved out of the vessel, thus also suggesting a migration defect. It has been shown in mouse that reducing *Vegfc*, *Flt4*, or *Prox1* function affects lymphatic development through a feedback loop that controls LEC precursor migration out of the vein^21,43^. Therefore, the reduced pool of lymphatic precursors we observed in *Hhex* mutants might be explained by the direct regulation of *Prox1* by HHEX, while the migration defects might be due to the downregulation of *Flt4*.

*Tie2-cre* mice have been shown to recombine in endothelial and hematopoietic cells which could also explain the blood-filled lymphatic phenotype we observed at later stages. To identify the direct effects of Hhex on LECs and avoid early defects in vascular development, we used a specific lymphatic *cre* line (*Prox1-cre*^*ERT2*^) and found that *Hhex* recombination caused defects in the formation of the lymphatics. Importantly, it has been reported that *Prox1-cre*^*ERT2*^ is most effective at late embryonic stages (from E12.5 in the skin)^44^, suggesting that the phenotype we observed in the skin is due to a late deletion of *Hhex*, and may explain why we observed LEC migration rather than specification defects. Determining the role of Hhex during early lymphangiogenesis will likely require the use of other *cre* lines in order to target earlier time points.

Importantly, our work has identified HHEX as a crucial transcriptional regulator of lymphangiogenesis and provides new insights into the transcriptional regulation of lymphatic differentiation and homeostasis. Lymphedema, a dysfunction of lymphatic vessels, has a worldwide incidence of 300 million cases and almost half of the cases have a genetic origin^45^. Yet, only very few causative mutations have been identified in these patients, making the identification of new candidate genes or signaling pathways regulating lymphatic development a crucial research interest^46^. Notably, most of the genes implicated in lymphatic pathologies are associated with the VEGFC/FLT4/PROX1 axis, which is essential for lymphatic physiology not only during development^1,3,31^ but also during tissue regeneration and cancer^47^. Therefore, the analysis of additional HHEX targets may help identify novel genetic causes of lymphedema, and also better modulate lymphatic vessels in various pathologies.

## Methods

### Zebrafish husbandry and strains

All zebrafish husbandry was performed under standard conditions in accordance with institutional (MPG) and national ethical and animal welfare guidelines. We used the following lines: *TgBAC(etv2:EGFP)*^*ci1*^ ^48^, *Tg(kdrl:NLS-mcherry)*^*is4*^ ^49^, *Tg(kdrl:EGFP)*^*s843*^ ^50^, *Tg(fli1a:nEGFP)*^*y7*^ ^51^, and *Tg(fli1a.ep:DsRedEx)*^*um13*^ ^52^, *TgBAC(prox1a:KalT4-UAS:uncTagRFP)*^*nim5*^ ^53^, and *Tg(-5.2lyve1b:DsRed)*^*nz101*^ ^54^. All experiments were performed using the *hhex*^*s722*^ allele.

### Imaging

Images were acquired using a Nikon SMZ25, a Zeiss Stereo Discovery V8, a Zeiss LSM780 confocal, or a Zeiss LSM800 confocal after embryo anesthesia with a low dose of tricaine and immobilization in 2% low-melting agarose in glass bottom petridishes (MatTek corporation). For time-lapse imaging, images were acquired using a Zeiss Cell Observer SD microscope. Embryos were mounted in 0.6% low-melting agarose and kept at 28 °C. Time-lapse images were recorded every 30 min. Facial lymphatics were measured using the ZEN software to determine the extent of formation of the different facial lymphatic vessels^55,56^.

### Generation of *hhex* mutants

*hhex* mutants were generated by TALEN mutagenesis targeting exon 3. TALENs were assembled using the Golden Gate method^13^ and 100 pg of total capped RNA per TALEN arm was injected into one-cell stage embryos. The following TAL effector domains RVDs were used: NI NG HD NG NN NG HD NG HD HD NN HD HD HD NN NI NN and HD NG HD NN HD NG NN NI NN HD NG NN HD NI NN HD NI NG HD NG.

Both alleles were genotyped by high-resolution melt analysis of PCR products. The primer sequences can be found in Supplementary Data 1.

### Quantifications

Quantification of vISVs and TD extensions was performed across somites located above the yolk sac extension.

### *FLT4* mRNA injections

Full-length human wild-type *FLT4* cloned into pCS2 plasmid^18^ was linearized using *Not*I. *FLT4* mRNA was then synthesized using a mMESSAGE mMACHINE SP6 Kit (Life Technologies) and purified using an RNA Clean and Concentrator Kit (Zymo Research). *FLT4* mRNA was injected at 300 pg per embryo at the one-cell stage.

### Whole-mount in situ hybridization

For whole-mount in situ hybridization^57^, embryos were fixed in 4% paraformaldehyde (PFA) overnight at 4 °C and subsequently dehydrated in methanol and stored at −20 °C until needed. In the first day, embryos were rehydrated to phosphate-buffered saline (PBS)/0.1% Tween-20 and then digested in 10 μg ml^−1^ proteinase K (Roche) followed by fixation in 4% PFA. Embryos were pre-incubated with hybridization buffer at 70 °C for 3 h and then incubated with digoxigenin (DIG)-labeled RNA antisense probes at 70 °C overnight. The next day, after washing, the embryos were incubated with alkaline phosphatase-conjugated anti-DIG antibody (Roche) at 4 °C overnight. The last day, after washing, the signal was visualized with NBT-BCIP staining solution (Roche). Primer sequences used to generate the probes for *hhex*, *flt4*^58^, and *vegfc*^59^ can be found in Supplementary Data 1.

### Transplantation experiments

Cells from *Tg(fli1.ep:DsRedEx)* wild-type donor embryos were transplanted at mid-blastula stages into host embryos derived from *TgBAC(etv2:EGFP); hhex*^*+/−*^ heterozygous incrosses. Successfully transplanted embryos were selected and imaged at 5 dpf. Quantification of vISVs and TD extensions was performed across four somites above the yolk sac extension.

### Transgenesis

To generate the *Tg(fli1a:tdTomato-2A-hhex)*^*bns136*^ line, tdTomato was fused to the *hhex* coding sequence separated by a self-cleaving viral 2A peptide sequence using Cold Fusion Technology (System Biosciences). To generate the line, 25 pg of Tol2 transposase mRNA and 25 pg of *pTol2-fli1a:tdTomato-2A-hhex* DNA were co-injected at the one-cell stage. The primers used for genotyping *hhex* mutants in the transgenic endothelial overexpression background can be found in Supplementary Data 1.

### Quantification and statistical analysis

No statistical methods were used to predetermine sample size. The experiments were not randomized. The investigators were not blinded to allocation during experiments or outcome assessment, except for the data shown in Figs. 2–4a, b. Representative images were selected from at least three independent experiments. Statistical analysis was performed using the GraphPad software. Values are presented as mean ± s.e.m. *P* values (*P ≤ 0.05, **P ≤ 0.01, ***P ≤ 0.001, ****P ≤ 0.0001) were calculated using Student’s *t*-test except for the qPCR (Mann–Whitney nonparametric test) and ChIP (Bonferroni post hoc) experiments.

### Mouse strains

All mice used were on a C57BL/6J background. The *Hhex*^*fl/fl*^^26^, *Tie2-cre*^28^, *Prox1-cre*^*ERT2*^ ^34^, *Cmv-cre*^27^, and *Hhex-IRES-Venus*^25^ mouse strains have been described. To generate *Hhex*^*+/−*^ mice, *Cmv-cre* females were crossed with *Hhex*^*fl/fl*^ males. Mice positive for *Cmv-cre* were then outcrossed with C57BL/6J mice to generate *Hhex*^*+/−*^ mice. Deletion of the *Hhex* locus was confirmed by PCR^60^. To generate embryos, *Hhex*^*+/−*^ males were crossed with *Hhex*^*+l−*^ females. *Tie2-cre Hhex*^*fl/+*^ males were crossed with *Hhex*^*fl/fl*^ females to generate embryos. The day of vaginal plug observation was considered as E0.5. *Prox1-cre*^*ERT2*^ animals were crossed with *Hhex*^*fl/fl*^ to generate *Prox1-cre*^*ERT2*^ *Hhex*^*fl/fl*^. Cre-mediated recombination was induced at E10.5, 11.5, and 12.5 by intraperitoneal tamoxifen injection (2.5 mg/25 g at 20 mg ml^−1^). Embryos were harvested at E16.5 for analysis. All mouse experiments were conducted in accordance with institutional (MPG) and national ethical and animal welfare guidelines.

### Immunostaining

To perform PROX1 immunostaining^23,61^, zebrafish embryos were fixed overnight in 4% PFA, washed in 100% methanol, incubated 1 h on ice in 3% H_2_O_2_ in methanol, washed in 100% methanol, and stored at −20 °C. Samples were then permeabilized in wash buffer (PBS/0.1% Tween/0.1%Triton), blocked in 10% goat serum/1% bovine serum albumin in wash buffer overnight at 4 °C, and incubated with Prox1 antibody (1:100, 102-PA32, Reliatech) overnight at 4 °C. Samples were then washed five times with wash buffer, followed by washes with maleic buffer (150 mM maleic acid/100 mM NaCl/0.001% Tween-20 pH 7.4), followed by blocking in maleic buffer containing 2% blocking reagent (Roche) for 3 h at room temperature, then incubated overnight at 4 °C with goat anti-rabbit IgG–horseradish peroxidase (Abcam, ab6721, 1:1000) for TSA signal amplification. Following washes with maleic buffer and PBS, samples were incubated for 3 h with TSA Plus Cyanine 5 reaction (Perkin Elmer) and washed with wash buffer several times over 1–2 days at room temperature.

To perform whole-mount immunostaining in mouse^62^, tissues were fixed overnight in 4% PFA and blocked overnight in blocking buffer (TSA blocking reagent, Perkin Elmer). Tissues were then incubated for 2 days at 4 °C with biotinylated antibodies in blocking buffer followed by biotin-streptavidin-horseradish peroxidase amplifications using the Vectastain-ABC Kit (Vector labs). For cryostat sections, E10.5 embryos were fixed overnight in 4% PFA, cryopreserved with 30% sucrose before immersion in OCT (Tissue-Tek), and stored at −80 °C. Ten micrometer sections were selected (between the eyes and the heart) to identify the bilateral cardinal veins and perform quantification of LECs. For the analysis of the dorsal dermal lymphatic network, E16.5 embryos were fixed for 2 h in 4% PFA before the dorsal skin was dissected and stained. Quantification of skin lymphatic vessels was performed using ImageJ. The antibodies used were PECAM-1 (1:100, MEC13.3, BD Biosciences), GFP (1:100, GFP-1020, Aves Labs), LYVE1 (1:100, 103-PA50, Reliatech), FLT4 (1:100, AF743, R&D Systems), and NRP2 (1:100, AF567, R&D Systems).

### Primary cells

HUVECs and HDLECs were obtained from Lonza, cultured with endothelial growth medium-2 (Lonza), and confluent cells were used at passage 6 or less. qPCR analysis of HUVECs showed very minimal expression of *PROX1*, *LYVE1*, and *PDPN*.

### siRNA-mediated knockdown

Cells were transfected with small interfering RNAs (siRNAs) using Opti-MEM and Lipofectamine RNAiMAX (Invitrogen)^63^. The target sequence of the two siRNAs directed against *HHEX* mRNA can be found in Supplementary Data 1.

### FACS sorting and RNA sequencing

For isolating endothelial cells from 48 hpf *Tg(kdrl:EGFP)* siblings and *hhex* mutants, embryos were selected based on the presence or absence of parachordal lymphangioblasts. Embryos were then rinsed in HBSS (Gibco) followed by dissociation in TrypLE express (Gibco) at 28 °C with repeated pipetting. Endothelial cells were isolated using a BD Aria sorter and sorted for EGFP^+^ or EGFP^+^/tdTomato^+^ signals (for *Tg(kdrl:EGFP*);*Tg(fli1a:tdTomato-2A-hhex))*. RNA was isolated using the miRNeasy Micro Kit (Qiagen) combined with on-column DNase digestion (DNase-Free DNase Set, Qiagen) to avoid contamination by genomic DNA. RNA and library preparation integrity were verified with BioAnalyzer 2100 (Agilent) and LabChip Gx Touch 24 (Perkin Elmer). 6 ng of total RNA was used as input for SMARTer^®^ Stranded Total RNA-Seq Kit–Pico Input Mammalian Kit (Clontech). Sequencing was performed on a NextSeq500 instrument (Illumina) using v2 chemistry, resulting in a minimum of 30M reads per library with 1 × 75 bp single end setup. The resulting raw reads were assessed for quality, adapter content, and duplication rates with FastQC (Andrews S. 2010, FastQC: a quality control tool for high-throughput sequence data, http://www.bioinformatics.babraham.ac.uk/projects/fastqc). Reaper version 13–100 was used to trim reads after a quality drop below a mean of Q20 in a window of 10 nucleotides^64^. Only reads between 30 and 150 nucleotides were cleared for further analyses. Trimmed and filtered reads were aligned vs. the zebrafish genome version DanRer10 (GRCz10.87) using STAR 2.4.0a with the parameter “--outFilterMismatchNoverLmax 0.1” to increase the maximum ratio of mismatches to mapped length to 10%^65^. The number of reads aligning to genes was counted with featureCounts 1.4.5-p1 tool from the Subread package^66^. Only reads mapping at least partially inside exons were admitted and aggregated per gene. Reads overlapping multiple genes or aligning to multiple regions were excluded. Differentially expressed genes were identified using DESeq2 version 1.62^67^. Only genes with a maximum Benjamini–Hochberg corrected *p* value of 0.05, and a minimum combined mean of five reads were deemed to be significantly differentially expressed. The Ensemble annotation was enriched with UniProt data (release 06.06.2014) based on Ensembl gene identifiers. The gene set enrichment analysis was performed using Metascape^68^ (www.metascape.org). The RNA-seq data can be accessed via the accession code GSE111963 (https://www.ncbi.nlm.nih.gov/geo/query/acc.cgi?acc = GSE111963). Validation of modulated genes was performed by qPCR on dissected trunks in *hhex* mutants, wild-type siblings, and *Tg(fli1a:tdTomato-2A-hhex)* embryos.

### Quantitative PCR

Total RNA was isolated using the RNA Micro Kit (Qiagen). RNA was reverse transcribed using the Transcriptor High Fidelity cDNA Synthesis Kit (Roche) according to the manufacturer’s instructions. qPCR reactions were carried out using TaqMan Probe Master or SYBR Green I Master (Roche). Primer sequences used in this study are provided in Supplementary Data 1.

### Chromatin immunoprecipitation

HUVECs (5 × 10^6^ cells per ChIP) were infected with Adenovirus *Myc-HHEX* or Adenovirus *GFP* control virus and ChIP experiments were done^69^ using a Myc antibody (CST #9B11, 3 μg/ChIP). qPCR was performed in quadruplicate. All data were normalized to PRM2 (control). The control region used is located in chromosome 18 where HHEX should not bind. Primers used can be found in Supplementary Data 1.

### Data availability

The authors declare that all data supporting the findings of this study are available within the article and its supplementary information files or from the corresponding authors upon reasonable request.

The RNA-seq data have been deposited in the NCBI’s Gene Expression Omnibus database under accession code GSE111963.

## Electronic supplementary material

Supplementary Information

Description of Additional Supplementary Files

Supplementary Data 1

Supplementary Movie 1

Supplementary Movie 2
