# Supplementary material for: HHEX is a transcriptional regulator of the VEGFC/FLT4/PROX1 signaling axis during vascular development
Source: Nat Commun. 2018 Jul 13;9:2704. doi: 10.1038/s41467-018-05039-1 (PMC6045644; doi:10.1038/s41467-018-05039-1)
Supplement: Supplementary file 1 — Supplementary Information [file 41467_2018_5039_MOESM1_ESM.pdf]

SupFig1

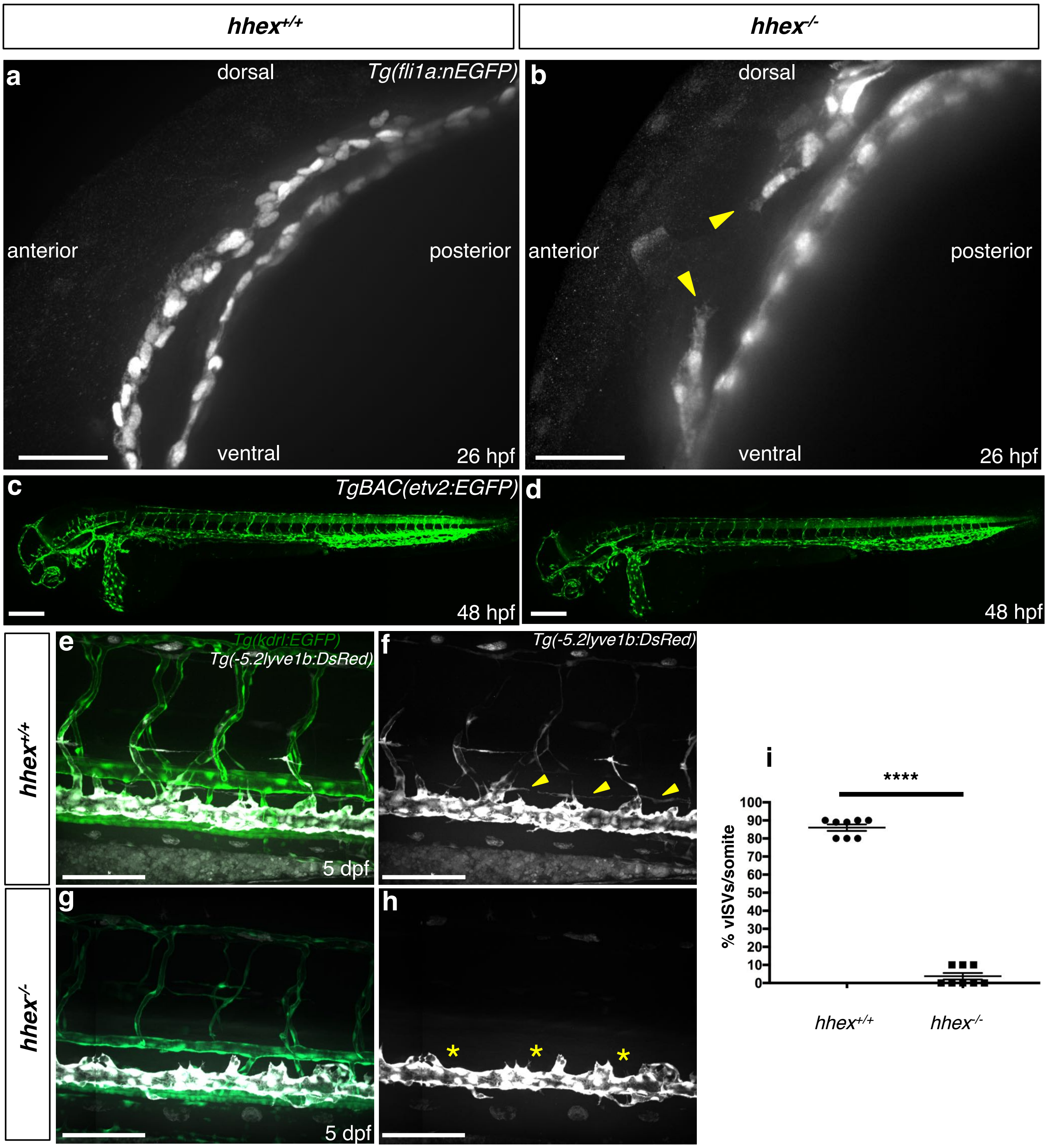

Supplementary figure 1: zebrafish *hhex* mutants exhibit a delay during PHBC formation but form arterial intersegmental vessels

(a-b) Maximum intensity projections of confocal images of 24 hpf *Tg(fli:nEGFP)*; *hhex*$^{+/+}$ and *hhex*$^{-/-}$ embryos after GFP immunostaining. *hhex* mutants exhibit delayed primordial hindbrain channels (PHBCs) formation (arrowheads). (c-d) Maximum intensity projections of confocal images of 48 hpf *TgBAC(etv2:EGFP)*; *hhex*$^{+/+}$ and *hhex*$^{-/-}$ embryos. *hhex* mutants form arterial ISVs. (e-h) Trunk vasculature of 5 dpf *Tg(kdrl:EGFP); Tg(-5.2lyve1b:DsRed); hhex*$^{+/+}$ and *hhex*$^{-/-}$. *hhex* mutants form arterial ISVs while vessels from the PCV (vISVs and TD) are mostly absent (arrowheads point to the ventrally positioned TD; asterisks indicate lack of this structure). i) Quantification of vISVs across 10 somites in 5 dpf *hhex*$^{+/?}$ (n=8) and *hhex*$^{-/-}$ (n=8). Values represent means ± s.e.m. ****$P \leq 0.0001$ by *t*-test. Scale bars: 100 µm (a-b), 200 µm (c-d), 50 µm (e-h).

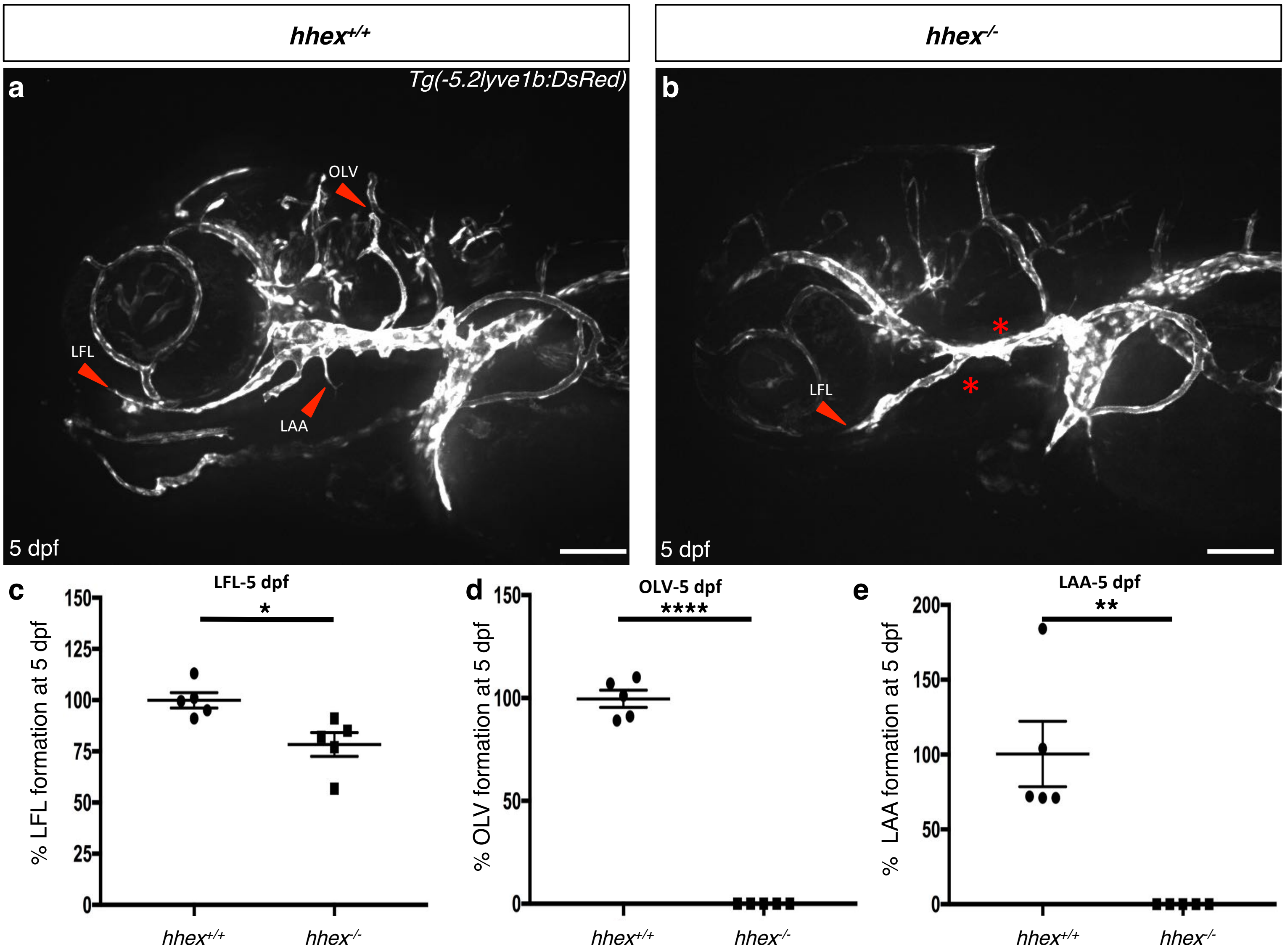

Supplementary figure 2: zebrafish *hhex* mutants exhibit facial lymphatic defects at 5 dpf
(a-b) Facial lymphatic network of 5 dpf *Tg(-5.2lyve1b:DsRed); hhex*$^{+/+}$ and *hhex*$^{-/-}$ larvae; *hhex*$^{-/-}$ exhibit defects in facial lymphatic vessel formation (arrowheads indicate facial lymphatics, asterisks indicate lack of these structures in *hhex* mutant). (c-e) Percentage formation of lateral facial lymphatic (LFL) (c), otolithic lymphatic (OLV) (d) and branchial arch lymphatic (LAA) (e) in *hhex*$^{+/?}$ (n=5) and *hhex*$^{-/-}$ (n=5). Values represent means ± s.e.m. ****$P \leq 0.0001$, **$P \leq 0.01$ and *$P \leq 0.05$ by *t*-test. Scale bars: 100 µm.

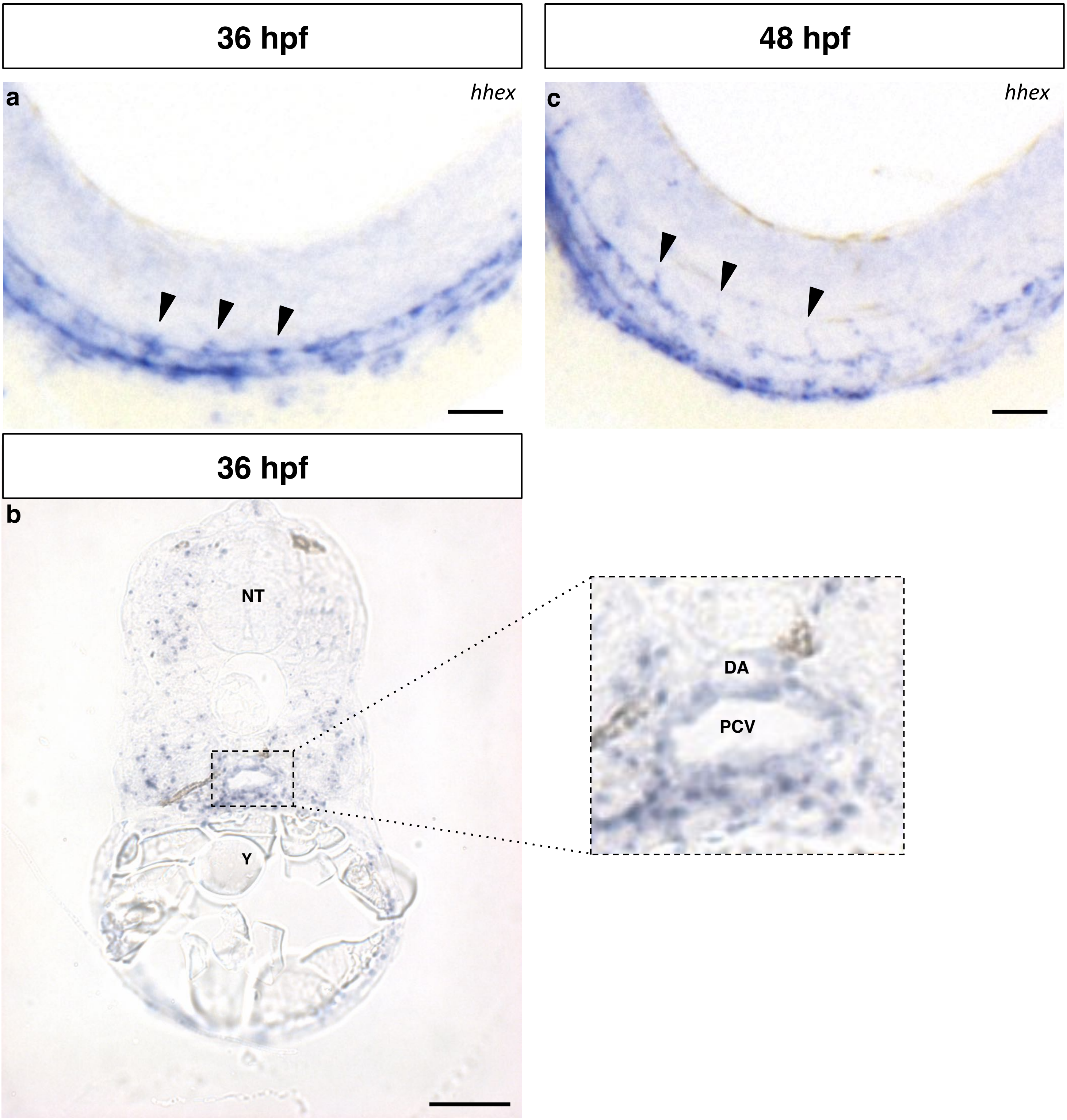

Supplementary figure 3: zebrafish *hhex* is expressed in endothelial cells sprouting from the PCV
hhex expression in the zebrafish trunk at 36 (a,b) and 48 (c) hpf by *in situ* hybridization. *hhex* is expressed by endothelial cells in the PCV and in vISVs (arrowheads). Transverse section through the embryo indicates expression in the PCV and in the ventral wall of the DA. Scale bars: 50 µm.

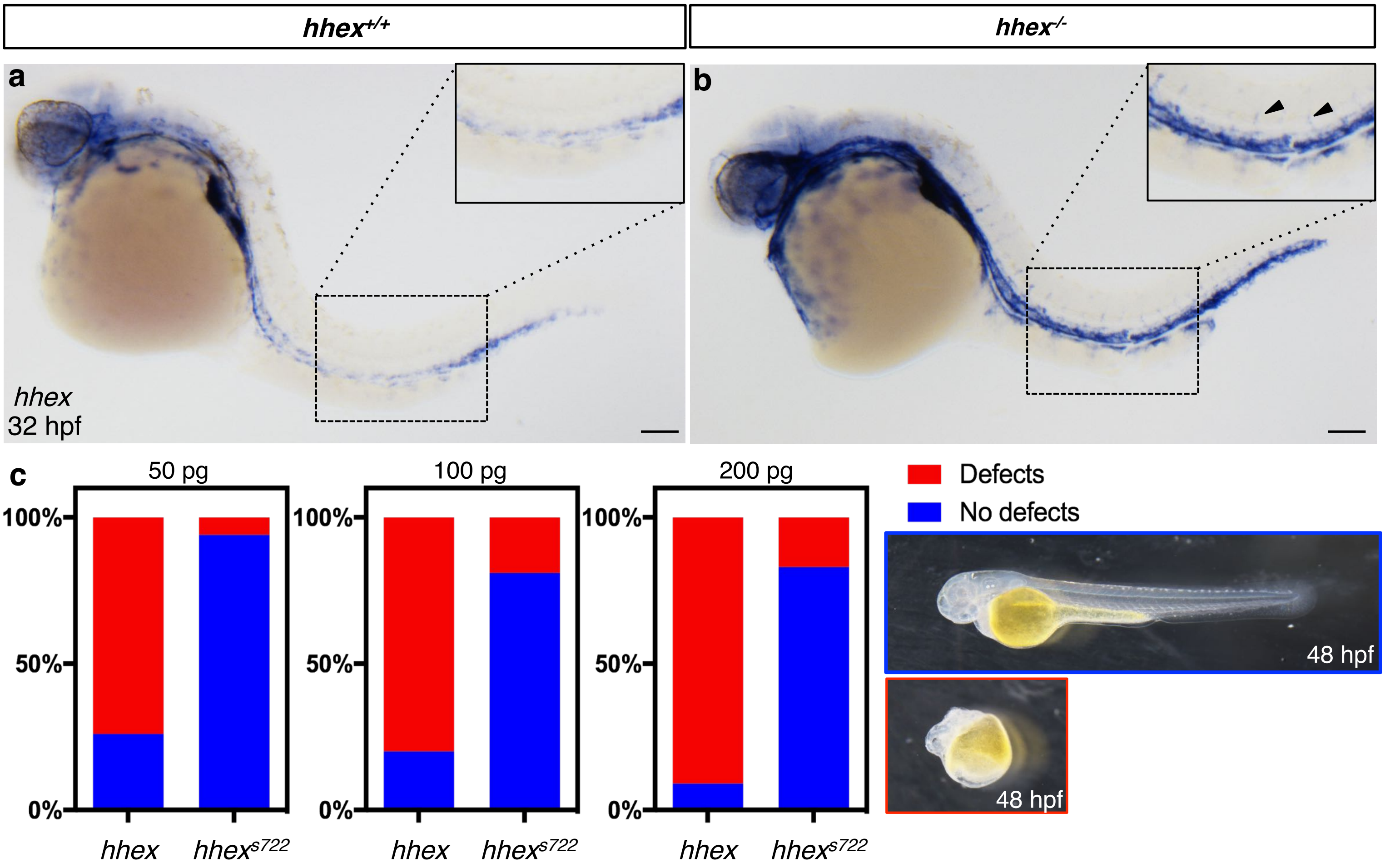

Supplementary figure 4: zebrafish *hhex* mutants exhibit increased *hhex* expression and the *hhex*^{s722} allele appears to encode a non-functional protein

(a-b) Whole-mount *in situ* hybridization showing *hhex* expression in 32 hpf *hhex*$^{+/+}$ and *hhex*$^{-/-}$ embryos. At 32 hpf, *hhex*$^{-/-}$ exhibit increased *hhex* expression in the vasculature (arrowheads point to ISVs). (c) *hhex* and *hhex*^{s722} mRNA injection at 50, 100 and 200 pg into one-cell stage wild-type embryos. Injection of wild-type *hhex* mRNA, but not *hhex*^{s722} mRNA, leads to developmental defects. Scale bars: 100 µm.

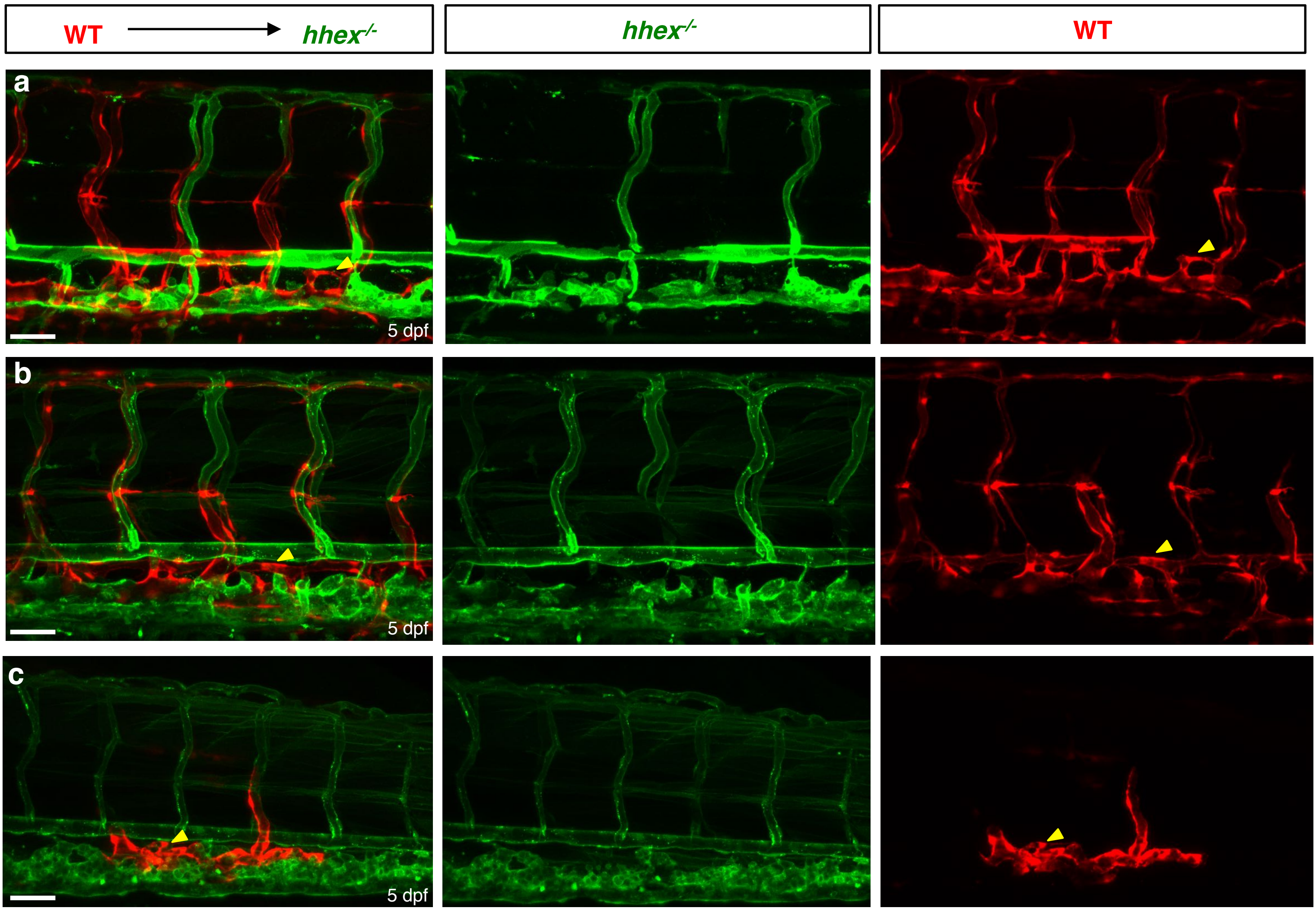

Supplementary Figure 5: Trunk vascular patterning in 3 different *hhex* mutants after tranplantation of WT cells.

(a-c) Transplantation of wild-type *Tg(fli1ep:DsRedEx)* donor cells into mutant *TgBAC(etv2:EGFP)* hosts derived from *hhex*$^{+/-}$ incrosses. Wild-type endothelial cells contribute to arteries, veins and lymphatics in *hhex* mutant hosts at 5 dpf (arrowheads point to TD). Scale bars: 50 µm.

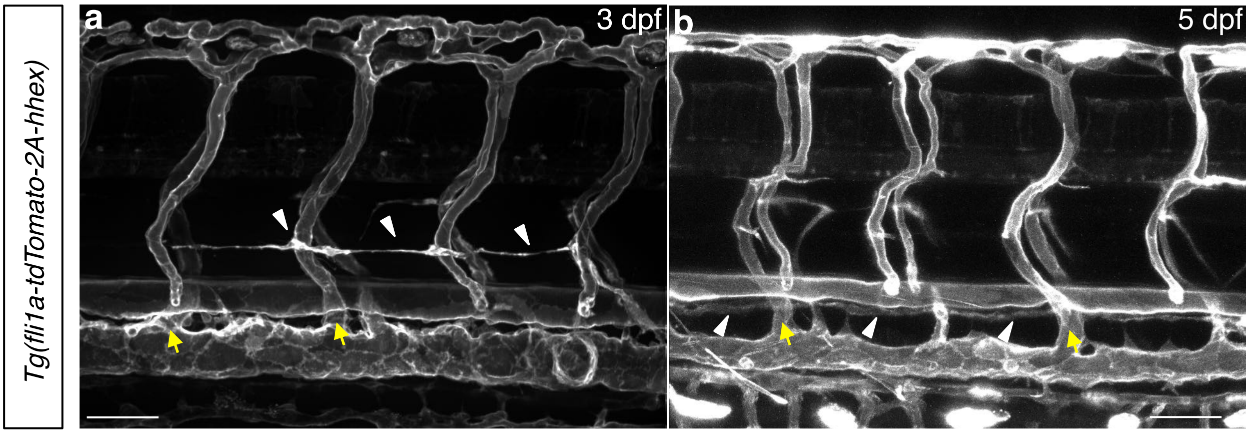

Supplementary figure 6: *Tg(fli1a:tdTomato-2A-hhex)* zebrafish embryos develop arteries, veins and lymphatic vessels

(a-b) Trunk vasculature of *Tg(fli1a:tdTomato-2A-hhex)* animals at 3 (a) and 5 (b) dpf. *Tg(fli1a:tdTomato-2A-hhex)* animals develop arterial, venous and lymphatic vessels (arrows point to vISVs; arrowheads point to parachordal lymphangioblasts (a) or TD (b)). Scale bars: 50 µm.

SupFig7

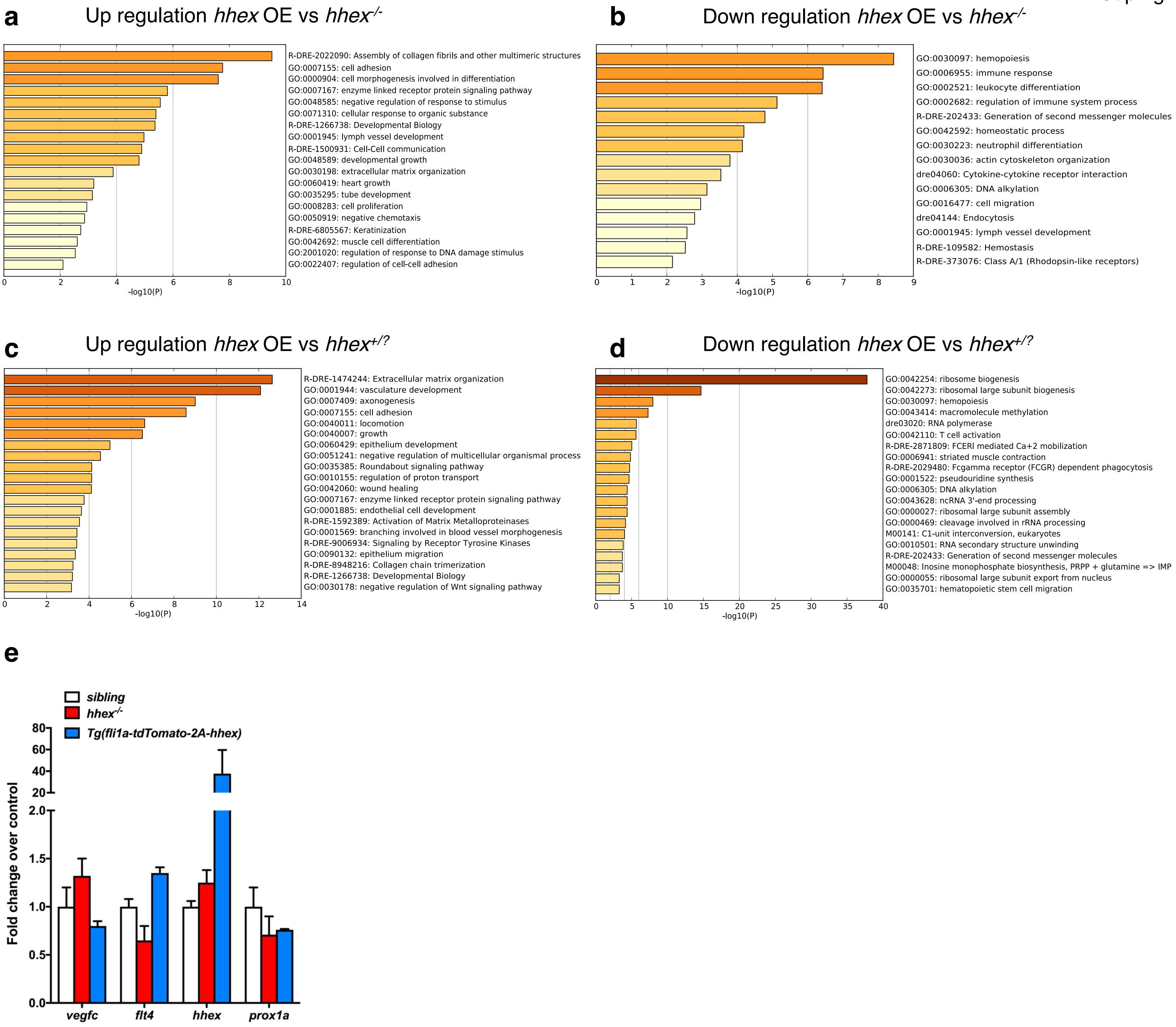

Supplementary figure 7: Gene set enrichment analysis and validation of the RNA sequencing data
(a-b) Gene set enrichment analysis of up (a) and down (b) regulated genes from 48 hpf FACS-sorted *hhex*-overexpressing endothelial cells compared to *hhex*$^{-/-}$ endothelial cells. Analysis of gene set enrichment highlights the role of *hhex* during lymphatic vessel (up regulation) and hematopoiesis (down regulation). (c-d) Gene set enrichment analysis of up (c) and down (d) regulated genes from 48 hpf FACS-sorted *hhex*-overexpressing endothelial cells compared to *hhex*$^{+/?}$. Analysis of gene set enrichment highlights the role of *hhex* during vascular development (upregulation) and hematopoiesis (downregulation). (e) qPCR analysis of *vegfc*, *flt4*, *hhex* and *prox1a* expression in *hhex*$^{+/?}$, *hhex*$^{-/-}$ and *hhex* overexpressing embryos shows that in *hhex*$^{-/-}$ compared to *hhex*$^{+/?}$ *vegfc* expression is upregulated while *flt4* and *prox1a* expression are downregulated; comparison between wild-type siblings and embryos where *hhex* is overexpressed in endothelial cells indicates that *vegfc* and *prox1a* expression are downregulated while *hhex* and *flt4* expression are upregulated.

SupFig8

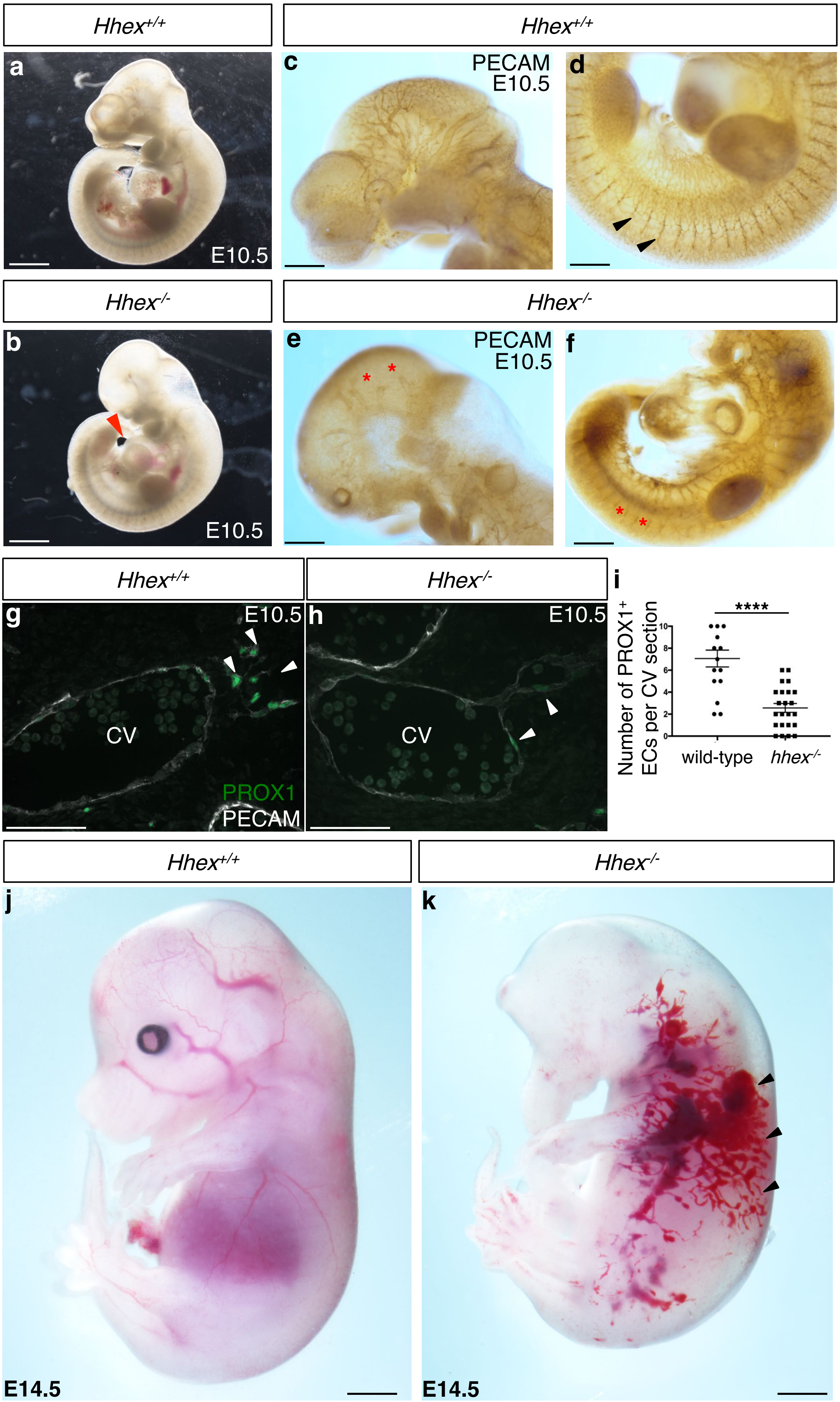

Hhex+/+
Hhex+/+
a
E10.5
c
PECAM
E10.5
d
Hhex-/-
Hhex-/-
b
E10.5
e
PECAM
E10.5
f
Hhex+/+
Hhex-/-
i
g
E10.5
h
E10.5
CV
CV
PROX1
PECAM
Number of PROX1+ ECs per CV section
****
wild-type
hhex-/-
Hhex+/+
Hhex-/-
j
k
E14.5
E14.5

Supplementary Figure 8: Mouse *Hhex*$^{-/-}$ embryos exhibit a strong vascular defect and a reduced number of PROX1^{+} endothelial cells at E10.5

(a-b) Whole-mount views of E10.5 *Hhex*$^{+/+}$ and *Hhex*$^{-/-}$ embryos. *Hhex*$^{-/-}$ exhibit pericardial edema (red arrowhead) as well as developmental delay. (c-f) Whole-mount views of E10.5 *Hhex*$^{+/+}$ and *Hhex*$^{-/-}$ embryos after PECAM immunostaining. *Hhex*$^{-/-}$ exhibit a strong blood vessel defect in the head and intersomitic vessel region (arrowheads point to intersomitic vessels in *Hhex*$^{+/+}$; asterisks indicate lack of vessels in the same region in *Hhex*$^{-/-}$). (g-h) Maximum intensity projections of confocal images from transverse cryosections of E10.5 *Hhex*$^{+/+}$ and *Hhex*$^{-/-}$ embryos after PECAM (white) and PROX1 (green) immunostaining in the CV region. *Hhex*$^{-/-}$ exhibit fewer PROX1^{+} /PECAM^{+} endothelial cells in the CV (arrowheads point to PROX1^{+} /PECAM^{+} endothelial cells). (i) Quantification of the number of PROX1^{+} endothelial cells in the CV region of E10.5 *Hhex*$^{+/+}$ (n=2) and *Hhex*$^{-/-}$ (n=2) embryos. (j-k) Whole-mount views of E14.5 *Hhex*$^{+/+}$ and *Hhex*$^{-/-}$ embryos. *Hhex*$^{-/-}$ exhibit edema and blood-filled lymphatics (arrowheads). Values represent means ± s.e.m. ****$P \leq 0.0001$ by *t*-test. Scale bars: 500 µm (a-b), 200 µm (c-f), 50 µm (g-h), 2 mm (j-k).

Supplementary Table 1: RNA sequencing data[1]

ID	*hhex*$^{+/?}$ Sample 1	*hhex*$^{+/?}$ Sample 2	*hhex*$^{-/-}$ Sample 1	*hhex*$^{-/-}$ Sample 2	*hhex* OE Sample 1	*hhex* OE Sample 2	*hhex*$^{+/?}$ vs *hhex*$^{-/-}$ p value	*hhex*$^{+/?}$ vs *hhex* OE p value	*hhex*$^{-/-}$ vs *hhex* OE p value
hhex	204	279	613	1157	764	925	0,009	9,51E-08	0,98
prox1a	439	880	156	322	541	747	0,41	0,96	0,052
prox1b	185	363	101	295	535	418	0,99	0,15	0,19
mafba	349	577	195	458	722	707	0,99	0,09	0,19
sox18	624	1332	563	1184	2463	2161	0,99	0,003	0,003
nr2f2	361	577	249	708	816	874	0,99	0,04	0,48
nrp2a	222	545	150	272	775	818	0,99	0,04	0,0007
nrp2b	783	1885	299	781	2946	3225	0,99	0,008	0,00001
flt4	1052	2166	302	1312	2815	3152	0,99	0,04	0,02
vegfc	80	171	108	209	108	88	0,99	0,72	0,64
stab1	2453	5117	1313	5144	8046	6824	0,99	0,02	0,24
stab2	1838	4333	1462	4674	7783	7152	0,99	0,003	0,14
ephb4a	509	900	410	1132	1288	1313	0,99	0,03	0,51
ephb4b	542	769	338	831	1231	1214	0,99	0,01	0,23
mrc1a	2111	4499	1644	5565	7322	6350	0,99	0,01	0,41
lyve1b	190	483	115	364	927	930	0,99	0,002	0,006
kdr	2350	3823	1325	4723	6804	5519	0,99	0,006	0,34
kdrl	9372	17884	7336	21007	25369	17167	0,99	0,19	0,66
cdh5	11023	16894	6545	19989	26660	20961	0,99	0,04	0,45
vegfaa	45	66	31	35	55	54	0,99	0,21	0,64
apln	719	1153	704	2481	433	504	0,99	0,01	0,02
aplnrb	113	285	79	306	447	582	0,99	0,01	0,16
adgrl4	521	626	272	845	1426	1009	0,99	0,002	0,26
esm1	536	821	361	1388	836	829	0,99	0,60	0,98
igfbp3	135	284	62	238	457	311	0,99	0,15	0,22
pfkfb3	380	262	164	192	628	404	0,99	0,19	0,01

1 Number of sequencing reads from 48 hpf FACS-sorted *hhex*$^{+/?}$ endothelial cells, *hhex*$^{-/-}$ endothelial cells and *hhex* OE endothelial cells (duplicates for each condition) and statistical analysis for all conditions (statistical significance indicated in red).
