# Supplementary material for: HHEX is a transcriptional regulator of the VEGFC/FLT4/PROX1 signaling axis during vascular development
Source: Nat Commun. 2018 Jul 13;9:2704. doi: 10.1038/s41467-018-05039-1 (PMC6045644; doi:10.1038/s41467-018-05039-1)
Supplement: Supplementary file 2 — Description of Additional Supplementary Files [file 41467_2018_5039_MOESM2_ESM.docx]

**Description of Additional Supplementary Files**

File Name: Supplementary Data 1

Description: List of primers used in this study.

File Name: Supplementary Movie 1

Description: Time lapse imaging of *Tg(kdrl:EGFP);* wild-type zebrafish embryo from 32 to 48 hpf.

File Name: Supplementary Movie 2

Description: Time lapse imaging of *Tg(kdrl:EGFP);* *hhex^-/-^* zebrafish embryo from 32 to 48 hpf.
